# Exposure radius of a local coal mine in an Arctic coastal system; correlation between PAHs and mercury as a marker for a local mercury source

**DOI:** 10.1007/s10661-021-09287-5

**Published:** 2021-07-21

**Authors:** Frits Steenhuisen, Martine van den Heuvel-Greve

**Affiliations:** 1 Arctic Centre, University of Groningen, Aweg 30, 9718 CW Groningen, the Netherlands; 2 Wageningen Marine Research, P.O. Box 77, 4400 AB Yerseke, The Netherlands; 3 Marine Animal Ecology, Wageningen University, P.O. Box 338, 6700 AH Wageningen, The Netherlands

**Keywords:** Mercury, PAH, Sediment, Biota, BSAF, Hierarchical clustering, Molecular diagnostic ratio

## Abstract

Mercury in the Arctic originates from emissions and releases at lower latitudes and, to a lesser extent, from local and regional sources. The relationship between mercury (Hg) and polycyclic aromatic hydrocarbons (PAHs) in sediment can be applied as an indicator of the mercury source. This research examines the Hg contamination gradient from a land-based coal mine to the surrounding coastal environment to quantify the impact of local sources. Total mercury and PAH (Σ_14_PAH) were measured in terrestrial and marine sediments as well as in marine biota. Samples were collected at the mine and two reference sites. Mercury and Σ_14_PAH concentrations in samples collected at the mine site were significantly higher than those at the reference sites. This was also found in the biota samples, although less pronounced. This work addresses the complexities of interpreting data concerning very low contaminant levels in a relatively pristine environment. A clear correlation between PAH and Hg concentration in sediment was found, although a large number of samples had levels below detection limits. PAH profiles, hierarchical clustering, and molecular diagnostic ratios provided further insight into the origin of PAHs and Hg, showing that signatures in sediments from the nearest reference site were more similar to the mine, which was not the case for the other reference site. The observed exposure radius from the mine was small and diluted from land to water to marine biota. Due to low contamination levels and variable PAH profiles, marine biota was less suitable for tracing the exposure radius for this local land-based Hg source. With an expected increase in mobility and availability of contaminants in the warming Arctic, changes in input of PAHs and Hg from land-based sources to the marine system need close monitoring.

**Graphical abstract:**

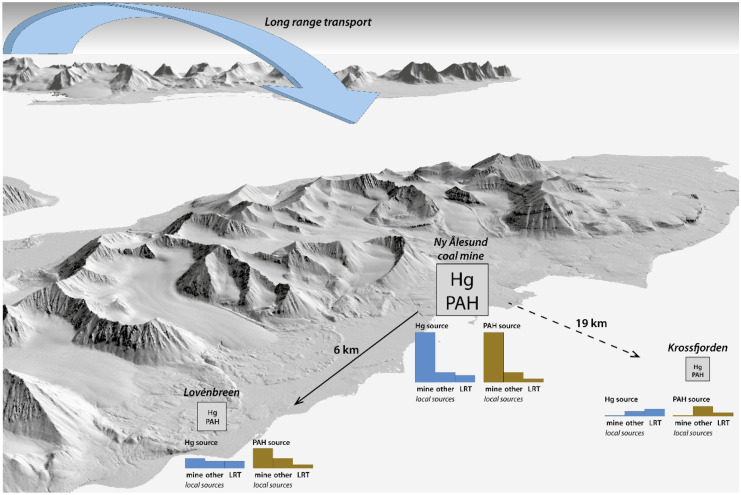

**Supplementary Information:**

The online version contains supplementary material available at 10.1007/s10661-021-09287-5.

## Introduction

Mercury (Hg) is a naturally occurring compound with toxic characteristics, posing risk to both humans and the environment (AMAP, 1998; AMAP/UN Environment, 2019, Gustin et al., 2016; Dietz et al., 2013). It can be found in highly variable concentrations in minerals and in fossil fuels, with coal in particular (AMAP/UN Environment, 2019). Mercury in coal originates from geogenic sources, having been assimilated from the atmosphere by the original plant species that formed the basis of coal. Mercury is emitted globally into the air and water through various natural and anthropogenic activities. For most of the Arctic with very low emissions from industrial activity (Steenhuisen & Wilson, 2019), the main source of (anthropogenic) mercury is long-range atmospheric transport (AMAP, 1998; AMAP, 2011; AMAP/UN Environment, 2019). In some areas of the Arctic, however, industrial activities such as mining and primary metal production occur, which may introduce mercury into the environment (AMAP, 2011; AMAP/UN Environment, 2019; Steenhuisen/Wilson, 2019)

Svalbard is an Arctic archipelago, situated north of Norway. On Svalbard, local sources of mercury can be attributed to the coal mining industry (both active and historical), the use of coal in two power plants and — to a lesser extent — waste and waste handling from settlements and shipping. While most mines on Svalbard have ceased or suspended operations (Ny-Ålesund, Pyramiden, Grumantbyen and most recently Svea), two mines are currently still in operation in 2020 (Longyearbyen and Barentsburg). At all sites, coal, mine tailings and (historic) industrial waste can be found on land. The historic coal mine of Ny-Ålesund is located at the southern shore of Kongsfjorden in the northwest of Svalbard. The mine was relatively small compared to the other mines on Svalbard and ceased operations in 1963 (Auen, 2016; Hanoa, 2016). Nevertheless, it has been related to mercury contamination as concentrations in the proximity of the mining area were reported to be elevated in soil, plants and biota when compared to reference sites (Van den Brink et al., 2018).

To assess the origin of mercury in the environment, polycyclic aromatic hydrocarbons (PAHs) have been applied in earlier studies (Liu et al., 2017), as mercury and PAHs often have common sources and pathways in the environment (Parsons et al., 2014). In the Yangtze River system, a moderate correlation was found between mercury and PAHs in sediments. This is however a complex system due to the fact that multiple sources of both PAHs and Hg are present (Liu et al., 2017). In the Kongsfjorden area, sources of mercury and PAHs are limited. Mercury and PAHs in soil and sediments in the mine area can originate from (1) coal present in the mine waste (through chemical (leaching) processes), (2) airborne deposition from local sources such as (historic) coal use, waste burning, diesel generators and shipping, and (3) from atmospheric deposition from long range transport of sources in other parts of Svalbard and further away. Coal has specific PAH profiles depending on its origin, with phenanthrene as the most dominant indicator compound (Achten & Hofmann, 2009).

Contaminants in coal mining piles and tailings can be transported from the original site to the marine ecosystem by freshwater runoff and melt water streams, both dissolved and bound to suspended particles. Parameters, such as the temperature regime and the presence of permafrost, can influence the chemical processes such as leaching and biodegradation of contaminants, and the subsequent run off (Colombo et al., 2018). The melt season on Svalbard starts around May/June and the ground is completely snow-free during summer (Maturilli et al., 2013). Current changes in the annual snow melt regime and permafrost leads to an earlier and longer snow free period and an increase in thickness of the active layer (Boike et al., 2018; Lameris et al., 2019). This is likely to affect the pathways through which mercury and PAHs enter and disperse into the terrestrial and marine environment.

This research examines the exposure gradient from a land-based mercury source, the historic Ny-Ålesund coal mine, to the surrounding environment. PAH profiles were used as an indicator to assess the exposure radius of Hg in this pristine Arctic environment. Mercury and PAH concentrations were therefore measured in sediments of meltwater streams in the mine area and at two reference locations, as well as in marine sediments and marine biota near the outflow of the meltwater streams. This study helps to infer how local sources contribute to mercury and PAH pollution in Svalbard (also in the context of long-range Hg transports from remote sources), and considers the complexities of interpreting data concerning very low contaminant levels in a relatively pristine environment.

## Materials and methods

### Sampling locations

**Fig. 1:**
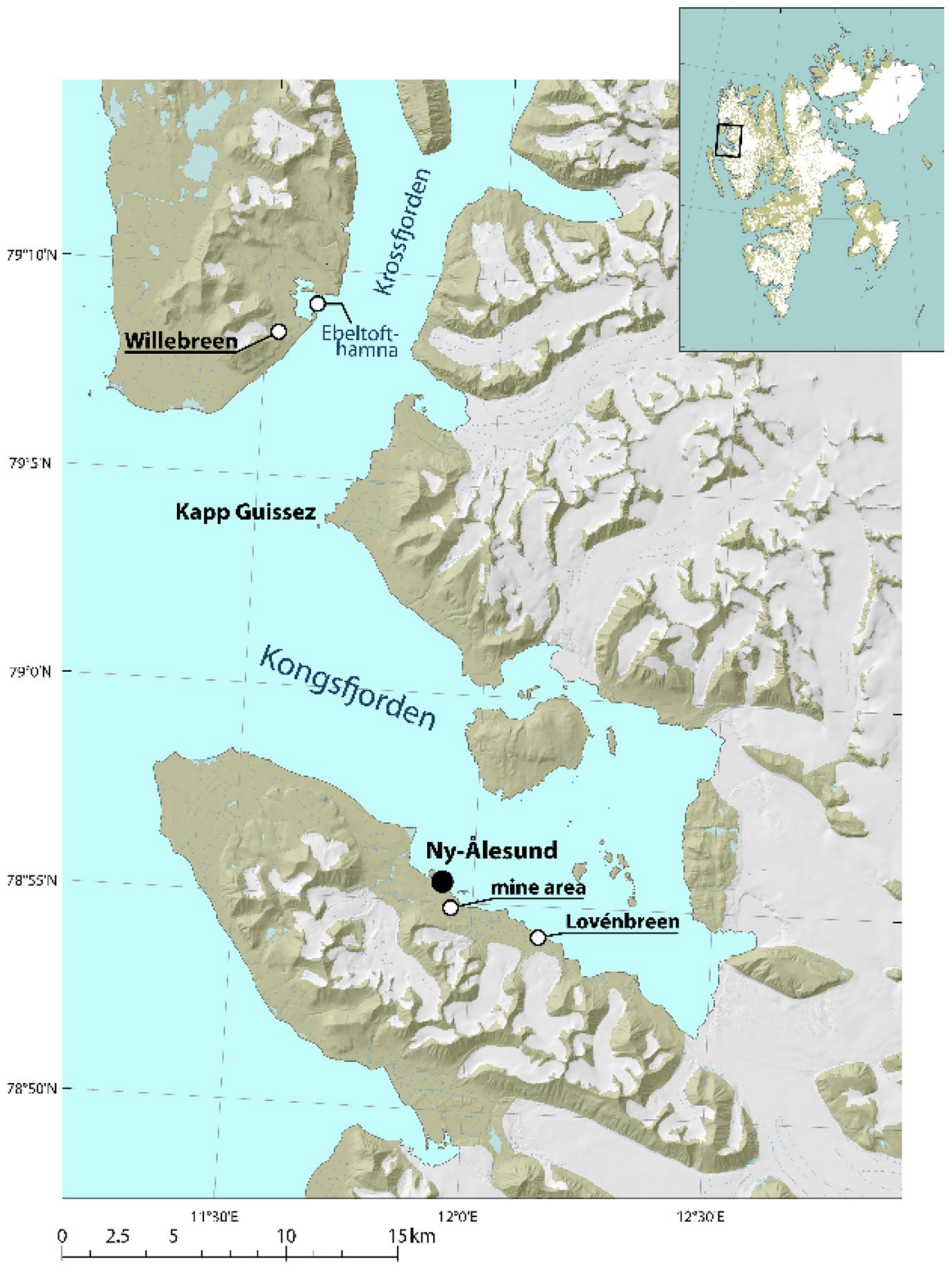
Sampling locations for this study. Terrestrial and marine samples collected in July 2017 in the Kongsfjorden and Krossfjorden, Svalbard

To estimate the magnitude and extent of local Hg sources, a variety of samples were collected in July 2017 at three sampling locations in the Kongsfjorden and Krossfjorden in northwest Svalbard (Fig. 1). The first one consisted of the historic coal mine which is located southeast of Ny-Ålesund. Two other areas served as Arctic reference sites. One is situated further to the inner fjord: the area below the Austre-Lovénbreen, approximately 6 km south-east of Ny-Ålesund and halfway towards the current (2017) glacier front of the Kronebreen to the east. Initially the area just north of Kapp Guissez in the Krossfjorden, approximately 16 km northwest of Ny-Ålesund, was selected as the second reference location. However, due to the lack of suitable sediments for sampling it was decided to shift to another location nearby. An alternative reference location was found below the Willebreen in Ebeltofthamna, located roughly at the same distance from Ny-Ålesund and with soil conditions better matching those at the other sampling sites.

At all sites, terrestrial sediment samples were taken near or in (dried up) meltwater streams. Marine sediment samples were collected near the outflow of the same streams just outside the tidal zone at a depth between ~ 12 and 52 m. The marine samples in the Krossfjorden were taken just outside Ebeltofthamna which was considered too shallow to provide samples comparable to the other locations. The marine samples near the Ny-Ålesund mine were taken outside the shallow area near the coast for the same reason. All marine sampling locations were outside the Kongsfjorden inner zone, which is described as being heavily influenced by large tidal glaciers, with a high influx of freshwater and glacial sediments (Hop & Wiencke, 2019, Svendsen et al., 2002; Duarte et al., 2019)

To infer the potential impacts of shipping on PAH concentrations in sediments, additional reference samples were collected at the Antarctic Peninsula in March/April 2019, a remote location without mercury sources nearby but with similar general environmental conditions (e.g., climate) as Svalbard. The samples were collected on Danco Island and Halfmoon Island (see map 1 in Supporting Information—SI). These locations are all frequently visited by ships comparable (and partly the same) to the ones which operate in Krossfjorden/Kongsfjorden area during the boreal summer.

### Sediment collection

Sediment samples were collected from the upper 5 cm of the sediment using a plastic corer (ø4cm PVC). Two samples were stored for chemical analysis; one sample for total mercury analysis in a 135-ml plastic jar, and one sample for PAH analysis in a 200-ml glass jar. Four sediments samples were collected per melt stream along a transect starting from upstream towards the shoreline. At the mine location, additional samples were collected upstream from the mine area, where the source of contamination was presumed to be below this point. Samples were stored at −20 °C within a few hours after collection until further processing. Marine sediments were collected using a Van Veen grab. Sediment samples were collected from the upper 5 cm of the sediment grab using a plastic corer and stored as specified above. In total, 37 terrestrial and 24 marine sediment samples were collected (see Table 2 in the SI). A piece of coal was collected at the Ny-Ålesund coal mine location to assess the Hg and PAH concentrations. The sample was stored in a plastic container at room temperature until further processing and homogenized prior to analysis.

### Biological samples

The local Hg and PAH contamination was also assessed in organisms to determine whether exposure to contaminants of the mine can be observed at the level of marine benthic organisms. Therefore, two marine worm species (*Nephtys* sp. and *Polycirrus* sp.) and three species of marine bivalves (*Astarte borealis*, *Serripes groenlandicus* and *Macoma calcarea*) were collected from the marine sediment samples. Sediment samples were sieved on-board with ambient seawater and the specimens were collected from the sieve. The number of collected species and total volume was not sufficient to analyze both Hg and PAHs at all sites. Therefore, a selection of biota samples was made for both Hg and PAH analysis (see Tables 3 and 4 in SI). Bivalve samples were kept 24 h in aerated water at 4 °C to depurate their gut prior to storage (Chapman, 2016). Samples were stored at −20 °C after collection until further processing.

### Chemical analysis

#### Total mercury

Total mercury was analysed in sediment and biota using an ISO17025:2005 accredited method based on US EPA 7473 (USEPA, 2007). In short, samples were dried and decomposed at high temperature. With a supply of oxygen, the volatiles were led to a catalyst tube, where oxidation took place and halogens, nitrogen and sulphur-oxides were removed. The residual destruction products were then led to an amalgamator, which converted mercury compounds into metallic mercury. The level of mercury was quantified using a flameless atomic absorption spectrometer. The samples were measured against a calibration curve, which was prepared from a certified standard (TraceCERT) obtained from Fluka and analysed in the same manner. A certified reference material, Oyster Tissue NIST1566B, was measured with each set of samples with satisfactory results. The method Limit of detection (LOD) and limit of quantification (LOQ) were 0.15 and 0.3 µg/kg respectively. The LOQ could differ slightly between samples depending on sample intake with an average LOQ of 0.54 µg/kg. Samples were not corrected for blanks but blanks were required to be below LOD.

#### PAH

PAHs were analyzed in sediment and biota according to De Boer et al. (2001) and Van den Heuvel-Greve et al. (2016). The procedure involved PAH extraction from the sediments with soxhlet (hexane/acetone 1:1). The extract was concentrated to 10 ml, cleaned over a silica gel-aluminium oxide column and, after addition of 1 ml of acetonitril, concentrated by evaporation to 1 ml of acetonitril. The PAH levels in the acetonitril solution were analysed by HPLC equipped with fluorescence detection. The following 14 PAHs were included in the Σ_14_PAH: acenaphthene, fluorene, anthracene, phenanthrene, fluoranthene, pyrene, benzo(a)anthracene, chrysene, benzo(b)fluoranthene, benzo(k)fluoranthene, benzo(a)pyrene, dibenz(a,h)anthracene, indeno(1, 2, 3-cd)pyrene and benzo(g,h,i)perylene (see Table 1 in SI for abbreviations).

The limit of quantification (LOQ) was defined as < 1.5 times the method blank, or the lowest used calibration point (whichever was highest). Samples were not corrected for blanks. Certified reference material NIST2974a (freeze dried mussel tissue) and method blanks were analysed with each set of samples. All results for blanks and reference materials were within normal limits. Recoveries were between 80 and 120%. Method LOD was defined as 0.5·LOQ.

### Dry weight

Dry weight was determined according to ISO 17,025:2005 accredited SOP 2.10.3.011 ‘Animal tissue: Determination of the level of moisture’. Samples of approximately 1 g were dried for 3 h at 105 °C after which the dry weight was determined gravimetrically.

### Organic matter

Total organic carbon (TOC) content was determined gravimetrically as loss on ignition after being exposed for 12 h at 550 °C.

### Lipid weight

The lipid-level determination was modified from the Bligh and Dyer (B&D) method (De Boer, 1988). Samples were extracted three times with a mix of chloroform, methanol and demineralized water. Lipid level was determined by weighing the residue after evaporation of the solvent.

### Data treatment

Hierarchical clustering was applied to explore the differences in individual PAH concentrations between the sediment samples in order to examine the dissimilarities between the samples. The dendrograms were based on the Euclidean pairwise distance metric and the ‘complete’ linkage algorithm (Smoliński et al., 2002, 2012).

Contaminant sediment concentrations were tested using a one-way ANOVA and a Tukey multiple pairwise comparison. ANOVA assumption on homogeneity of variance and normality were met for most datasets. The data for Σ_14_PAH concentrations in terrestrial sediments did not fully meet the normality criterium. As the Kruskal–Wallis test revealed similar results as the Tukey multiple pairwise comparison, the Tukey test was considered valid.

To further identify PAH sources, the PAH molecular diagnostic ratios (MDRs) were applied (Tobizewski et al., 2012). This technique has proven its value to identify PAH sources in areas with moderate or high contamination levels. The high number of measurements close to, or below the detection limit, severely restricted the potential of MDR. Only two MDRs could be properly calculated: fluoranthene/pyrene (calculated as FLA/(FLA + PYR)) and ∑LMW/∑HMW, where ∑LMW is the sum of two and three-ring PAHs and ∑HMW is the sum of four and five-ring PAHs (Zhang et al., 2008). To visualise the relative uncertainty of the calculated PAH ratios, the markers in the ratio plots were scaled based on the number of measurements below DL, ranging from 0 (all 4 measurements < dl) to 4 (all 4 measurements > dl). The relative uncertainty per axis was indicated by horizontal and vertical markers which are scaled 0–2.

Biota–sediment accumulation factors (BSAF) for individual PAHs were calculated for *M. calcarea* as BSAF = (Corg/fLIP)/(Csed/fTOC), with Corg being the chemical concentration in the organism in wet weight (ww;μg/kg), Csed the chemical concentration in sediment in dry weight (dw;μg/kg), fLIP the fraction of lipids (%) in the organism based on wet weight (ww), fTOC the fraction of TOC (%) in sediment based on dry weight (dw) (Ankley et al., 1992). For sediment the average value per location (Ny-Ålesund mine, Lovénbreen and Krossfjorden) was used for both individual PAH compounds and TOC %. The BSAF for Hg was calculated based on dry weight concentrations in both organism and sediment (Corg/Csed).

## Results and discussion

### Sediment Hg and PAH concentrations

#### Terrestrial sediments

**Fig. 2:**
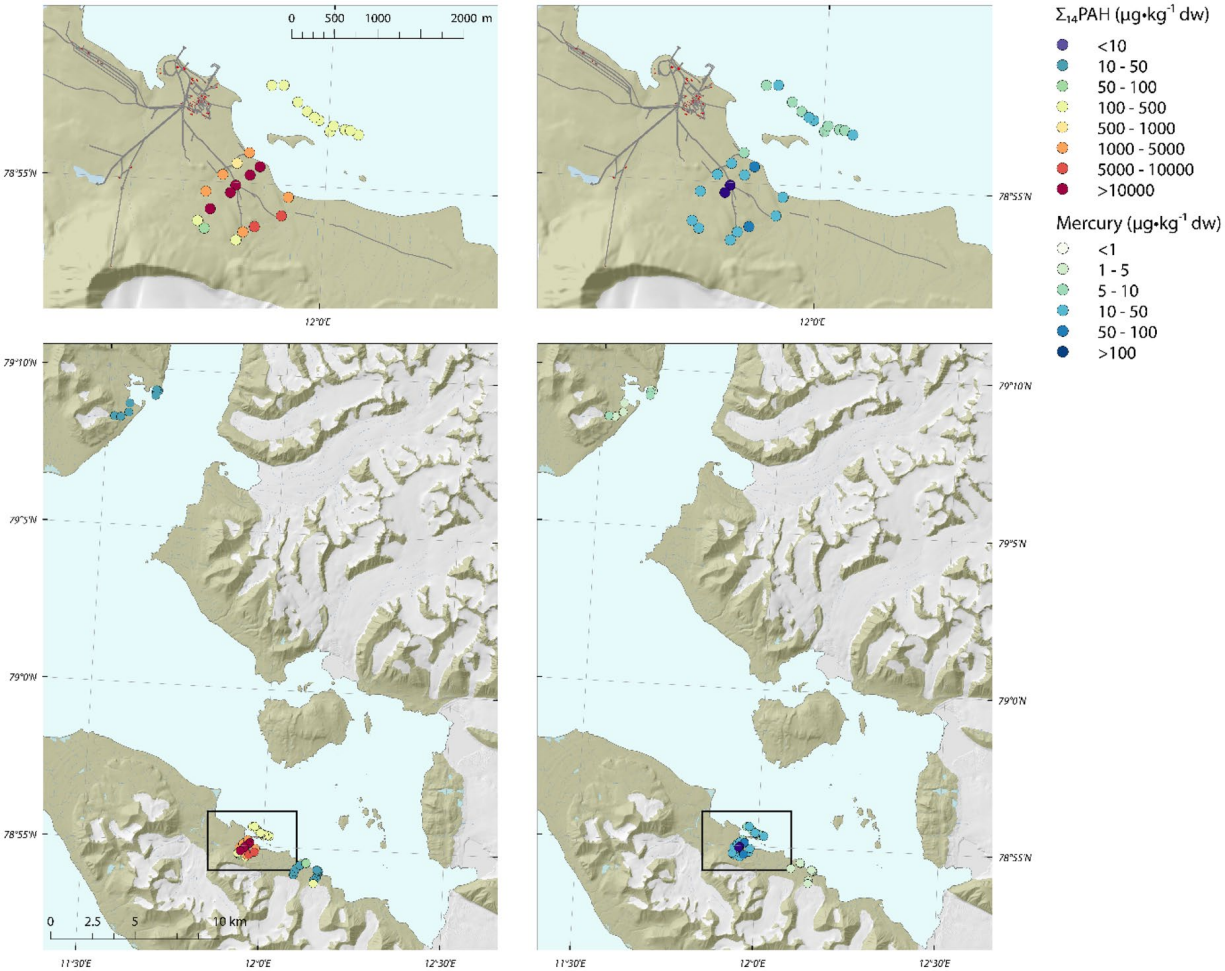
Σ_14_PAH (left) and mercury (Hg) (right) concentrations in terrestrial and marine sediments of Kongsfjorden and Krossfjorden, sampled in July 2017. PAH concentrations below detection limit were included in this graph as 0.5·dl

**Fig. 3:**
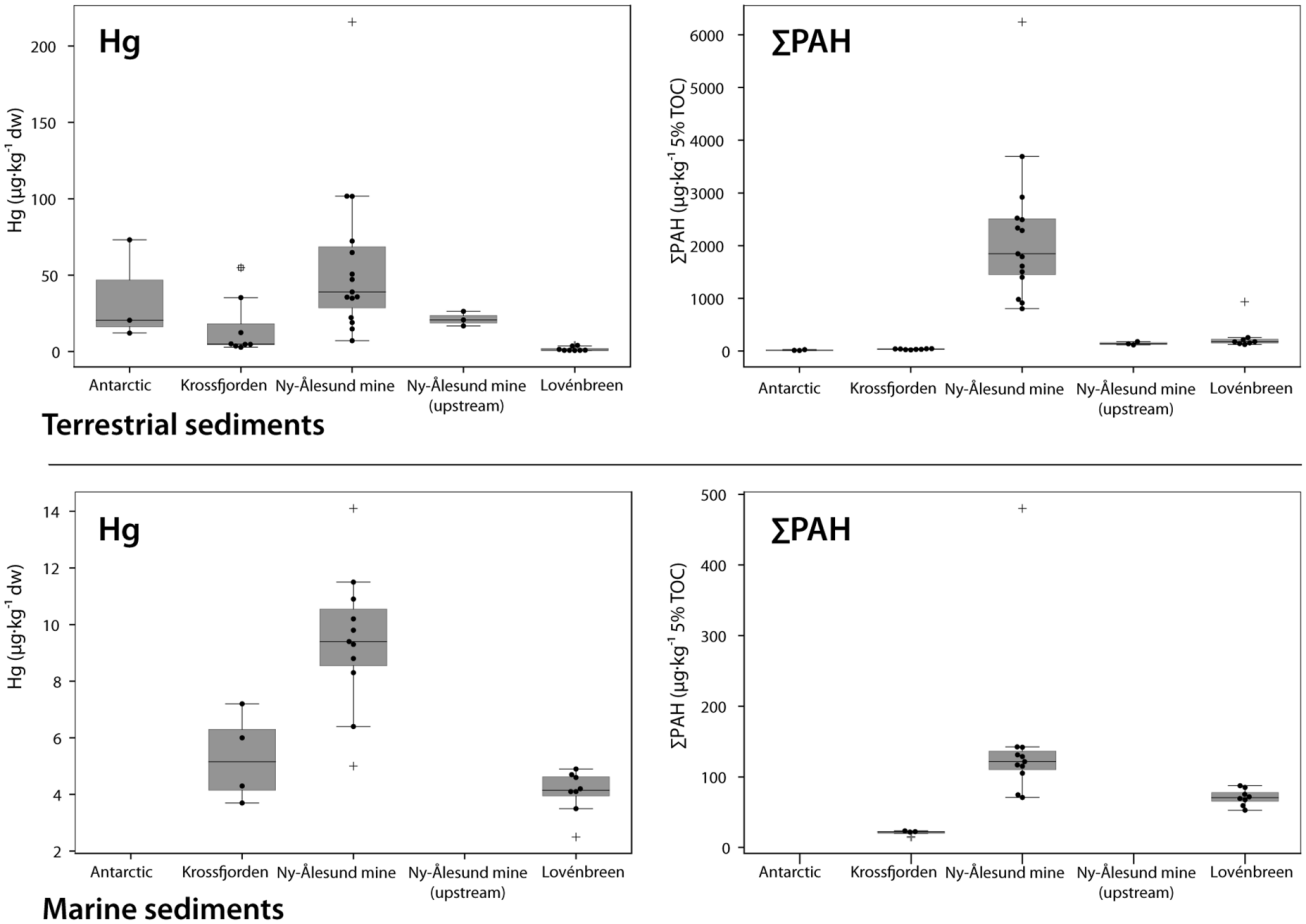
Boxplot of PAHs and Hg concentrations in terrestrial (upper graphs) and marine (lower graphs) sediment samples collected in 2017 in the Kongsfjorden and Krossfjorden, and in 2019 at the Antarctic Peninsula. Concentrations are in μg/kg 5% TOC for ΣPAH and μg/kg dw for Hg. Outliers are marked with + . Individual measurements are shown as dots. PAH concentrations below detection limit were included in this graph as 0.5·dl

The highest mercury concentrations were found in sediments from meltwater streams running through the Ny-Ålesund mine area (Fig. 2 and 3). Samples contained on average 58 ± 52 µg/kg dw Hg (range 7–216 µg/kg dw) and were significantly higher (*p* < 0.05) than the concentration found at the Lovénbreen and Krossfjorden reference sites (< 5 µg/kg dw) (Fig. 3). The Hg concentrations in the meltwater stream sediments in the mine correspond well with concentrations in soil samples (40–900 µg/kg dw) from the centre of the mine waste area (Van den Brink et al., 2018). This implies that the central area of the mine contains even higher Hg levels than the sediments collected in the meltwater streams running through the mine.

Mercury concentrations in three samples collected at the ‘clean, unaffected’ upstream area above the mine contained 17–26 µg/kg dw Hg, a factor 10 lower than the highest concentrations in the mine area and a factor two lower than the average concentration in the mine. The mercury observed in these samples most likely originated mainly from long-range atmospheric transport, and local air emissions (i.e., local use of coal and emissions from shipping) and other deposition processes. Hg deposition by long-range transport in the study area was estimated to be 4–8 µg/m^2^/year (modelled for year 2015, AMAP/UN Environment, 2019).

The mercury concentrations in the Antarctic samples (range 12–73 µg/kg dw, average 35 µg/kg dw) were higher than the reference locations on Svalbard and within the range of the concentrations found in the mine (Fig. 3). This is probably due to local geology, although local use of fossil fuels by research stations cannot be excluded at this point.

The Hg levels in terrestrial sediments found at the reference sites of this study were 2.6 ± 1.8 (0.07–5) µg/kg dw, based on the samples taken at Krossfjorden (*n* = 4) and Lovénbreen (*n* = 8). These are a result of the (highly) variable Hg presence in the local geology and soil composition as well as from Hg deposition from long range atmospheric transport. Several studies present average (background) Hg concentrations in soil on Svalbard. Halbach et al. (2017) report an average of 111 ± 36 Hg μg/kg in top soil and 25 ± 13 μg/kg in mineral soil. Gopikrishna et al. (2020) report a lower concentration of 40 ± 20 Hg μg/kg for topsoil. Both these studies report levels matching the concentrations that were found at the Ny-Ålesund mine site in our study, whereas the Hg concentrations in the reference areas were considerably lower. There are several reasons by which these differences can be explained. Both Halbach et al. (2017) and Gopikrishna et al. (2020) analysed samples collected in the vicinity of Ny-Ålesund, whereas Halbach et al. (2017) also measured in and near Longyearbyen (Halbach, 2016). These samples were collected at locations that were more exposed to Hg emissions than samples collected at more remote locations. Also, organic matter content in soil has been shown to strongly influence mercury concentrations with increasing organic matter content enhancing the ability of soil to retain Hg after deposition (Halbach et al., 2017). Average background mercury concentration in soil on Svalbard based on these samples may therefore be overestimated. Hg background concentration in soil of other remote (Arctic) locations was more in line with what we found in the Lovénbreen samples. Jiang et al. (2011) measured a range of 0.7–6.6 μg/kg dw in soil samples collected at a location just NW of Ny-Ålesund. A study in Greenland (Riget et al., 2000) reported Hg levels of < 10 Hg μg/kg dw in soil, being below or just above the detection limit. We therefore believe that the Hg levels measured at the Lovénbreen and Krossfjorden provide a good indication of the local background level to assess the exposure radius of the mine.

Total PAH concentrations in stream sediments showed a similar picture as Hg. The highest PAH concentrations were found in and around the mine site (Figs. 2 and 3) with an average concentration of 2222 ± 1321 μg/kg Σ_14_PAH, normalized on 5% TOC, and a range of 803–6244 μg/kg ΣPAH 5% TOC, for all mine samples except the three upstream samples. In several samples we observed a high density of coal particles and consequently a high TOC (up to 51%). These samples showed relatively high PAH levels, in some cases higher than the PAH levels found in the coal sample. This may be explained by the fact that a lump of coal on average may have a lower PAH concentration than coal-dust (particles) (NGI, 2003 and NGI, 2020). Σ_14_PAH concentrations were significantly higher than all other sites (*p* < 0.05). The average total PAH concentration found in the three upstream samples above the mine was lower with 143.4 ± 25 μg/kg Σ_14_PAH5% TOC (range 116–177 μg/kg 5% TOC). At the Lovénbreen reference site, total PAH levels were lower than those near the mine (average 271 ± 253 μg/kg Σ_14_PAH 5% TOC, range 123–933 μg/kg Σ_14_PAH 5% TOC), though slightly higher than those of the upstream mine samples. The Krossfjorden reference site showed the lowest total PAH levels of the Arctic samples (31–43 μg/kg Σ_14_PAH 5% TOC). The samples collected at the Antarctic Peninsula showed the lowest PAH concentrations of all, with 9–25 μg/kg Σ_14_PAH 5% TOC (Fig. 3).

No exceedance of guideline limits was found for mercury (1000 μg/kg dw), whereas in 12 out of 18 samples collected at the mine site, Σ_14_PAH exceeded the threshold of 2000 μg/kg dw for Σ_16_PAH in soil (NGI, 2020).

### Marine sediments

Mercury concentrations in the marine sediment samples collected near the mine area had an average Hg concentration of 9 µg/kg dw ± 0.002 (range 5–14 µg/kg dw) (Figs. 2 and 3). Both reference locations had significantly (*p* < 0.05) lower concentrations: Lovénbreen 4 µg/kg dw ± 0.001 and Krossfjorden 5 µg/kg dw ± 2.

The Σ_14_PAH concentration in the marine sediments near the mine showed a similar picture, with an average concentration of 148 ± 107 μg/kg 5% TOC (range 71–480 μg/kg Σ_14_PAH 5% TOC) (Figs. 2 and 3). The marine sediment samples taken near the Lovénbreen were lower and contained an average concentration of 71 μg/kg ± 11 μg/kg Σ_14_PAH 5% TOC (range 53–87 μg/kg Σ_14_PAH 5% TOC). Marine sediment samples collected at the Krossfjorden marine site had Σ_14_PAH concentrations between 15 and 23 μg/kg 5% TOC. All locations differed significantly in Σ_14_PAH concentrations (*p* < 0.05).

Even though marine sediment samples near the run-off from the mine showed higher Hg and PAH concentrations than the marine samples taken at the two reference sites, the contaminant signal was weaker than that in the terrestrial sediments. This can be explained by the fact that terrestrial stream sediments will be contaminated through leaching processes directly from the mine as well as atmospheric deposition. The marine system however will be influenced by dilution processes, tidal currents in the fjord and sediment load coming from the Kongsfjorden and Kongsbreen glacier system (Beldowski et al., 2015).

Mercury concentrations in the marine samples near the Ny-Ålesund mine (5–14 µg/kg dw) were slightly lower than reported in earlier studies in the Kongsfjorden (8–80 µg/kg dw; Beldowski et al., 2015; Grotti et al., 2013; Lu et al., 2012) and in the Grønfjorden (Barentsburg) (7–42 µg/kg dw, with levels increasing toward the inner parts of Grønfjorden; Lebedeva et al., 2018). The mine in Barentsburg is still operational and has had an overall higher coal production than the Ny-Ålesund mine. On top of that, the inner fjord of Grønfjorden may be influenced by deposition of Hg from the coal-burning power plants of nearby Barentsburg and Longyearbyen. The contribution from long-range atmospheric transport to Hg concentration found in this fjord is expected to be similar to that found in Kongsfjorden and Krossfjorden.

On a wider scale, mercury concentrations in the marine sediments were found to be comparable to other Arctic areas, such as the Chukchi Sea (5–55 µg/kg dw total Hg; Fox et al., 2014), Bering Sea (deep water < 2000 m; 20–36 µg/kg dw total Hg; Iricanin & Trefry, 1990), and Beaufort Sea (3–97 µg/kg dw total Hg; Trefry et al., 2003), and lower than reported for the north-eastern Bering Sea (< 1–130 µg/kg dw total Hg; Nelson et al, 1975) and Arctic Ocean Basin (34–116 µg/kg dw total Hg; Gobeil et al., 1999).

Hg concentrations in sediment were well below lowest reported no observed effect concentrations (NOEC; 2–551 mg/kg dw) and lowest observed effect concentrations (LOEC; 7–972 mg/kg dw) for Hg in marine and estuarine sediments (Conder et al., 2014). PAH levels in marine sediment from the Kongsfjorden from this study (36–182 μg/kg Σ_14_PAH dw) were on the lower side compared to other studies in this fjord (12–2315 μg/kg Σ_13_PAH dw, Szczybelski et al., 2016; 1–2550 μg/kg Σ_16_PAH dw, Van den Heuvel-Greve et al., 2016; 52–1482 μg/kg Σ_12_PAH dw, Pouch et al., 2017). Higher PAH concentrations were observed in samples collected near the settlement of Ny-Ålesund, which is influenced by a variety of PAH sources such as shipping, run-off from waste handling and treatment, as well as (historic) coal processing. Σ_14_PAH concentrations in our study were well below the sediment guideline values (class 2) of 2000 µg/kg Σ_16_PAH (Bakke et al., 2010).

The coal sample from the mine area contained 133 µg/kg dw Hg and 554 μg/kg 5%TOC Σ_14_PAH.

### PAH profiles

**Fig. 4:**
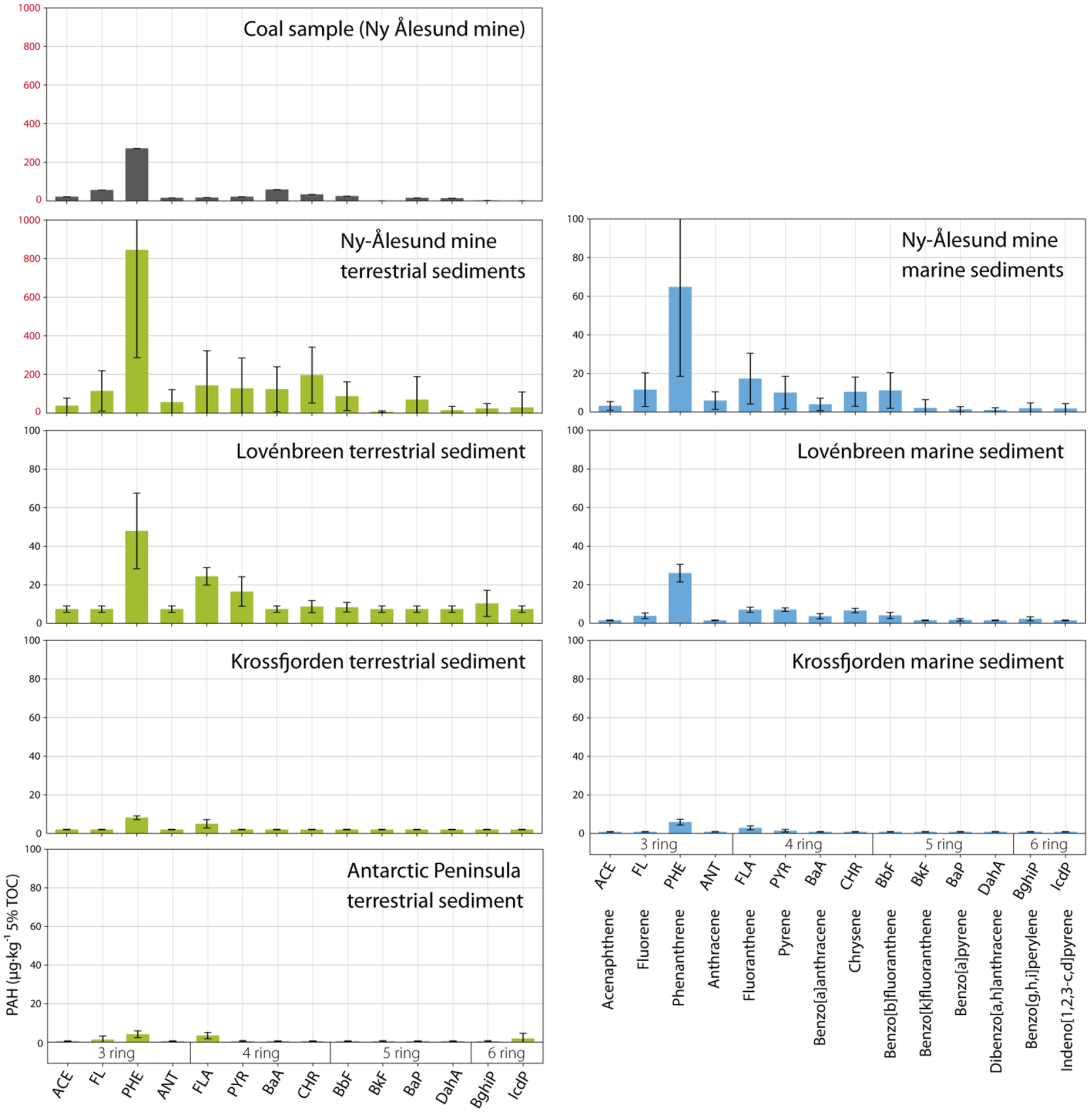
Average PAH profiles in sediment samples per location (μg/kg 5%TOC), collected in the Kongsfjorden and Krossfjorden in July 2017 and the Antarctic Peninsula in March/April 2019. Note the different y-axis scale for the Ny-Ålesund coal and terrestrial sediments (black = coal collected at Ny-Ålesund mine, green = terrestrial melt water stream sediment, blue = marine sediment). A deviating sample (terrestrial sediment from Lovénbreen) was excluded from this graph because of an extreme phenanthrene peak (414 μg/kg 5%TOC)

Average PAH profiles in terrestrial samples of Ny-Ålesund mine and the Lovénbreen, as well as the marine sediment near the mine were rather similar to the coal sample collected at the Ny-Ålesund mine (Fig. 4). All samples from these locations contained a clear phenanthrene peak, which has been described before as the most dominant PAH in coal, depending on the origin (Ribeiro et al., 2012). Phenanthrene dominance in Kongsfjorden marine sediment has also been observed in an earlier study, with an increasing occurrence from outer fjord towards the Kronebreen (Pouch et al., 2017). Pouch et al. (2017) however did not sample close to Ny-Ålesund and although providing an overall picture for the Kongsfjorden, they did not register the local elevated concentrations related to the mine. The Krossfjorden sediments (both terrestrial and marine) showed a low peak for phenanthrene while other compounds remained mostly below detection level. Very low PAH concentrations were observed in the terrestrial sediments from the Antarctic Peninsula.

### Origin of PAHs in terrestrial and marine sediments

#### Terrestrial sediments

**Fig. 5:**
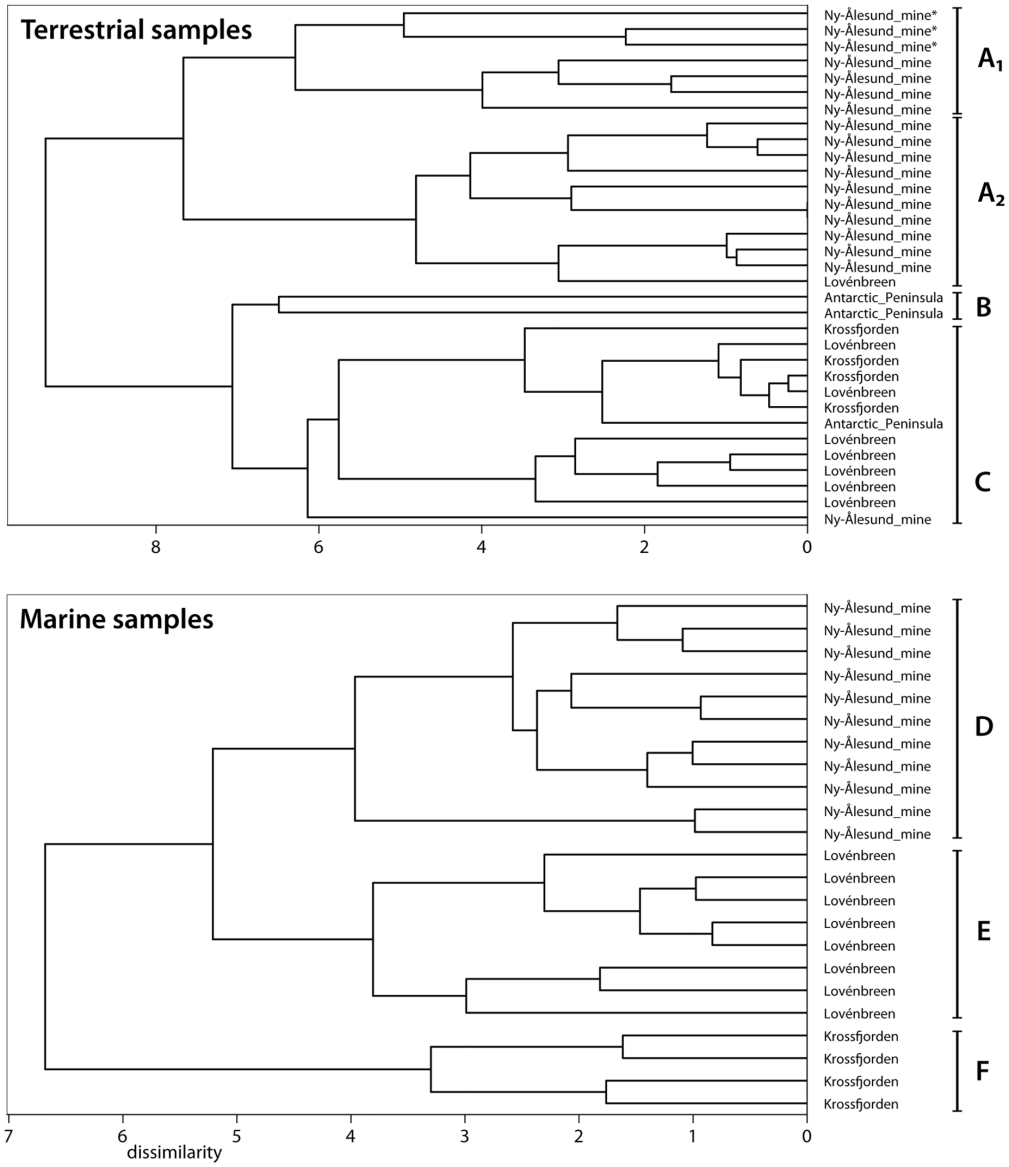
Dendrograms based on the PAH profiles of all samples (normalised on μg/kg 5% TOC). Top: The terrestrial sediment samples that were collected in the Kongsfjorden (2017), Krossfjorden (2017) and Antarctica (2019). Bottom: The marine sediment samples that were collected in the Kongsfjorden (2017) and Krossfjorden (2017). Three upstream samples from the mine area are labelled with *

Hierarchical clustering of TOC normalised PAH concentrations in the terrestrial sediment samples revealed four main clusters (Fig. 5). All except one sample from the mine area were in cluster A1 and A2. Cluster A2 also included one sample from the Lovénbreen area. The Lovénbreen and Krossfjorden samples formed cluster C. Two of the samples taken at the Antarctic Peninsula formed cluster B. The two top level clusters (A and [B,C]) separated the samples which were contaminated by both direct leaching from the mine waste as well as deposition from air (A1 and A2) from the samples which were contaminated by deposition from air only (B and C). Exceptions were three samples taken just upstream from the mine area which we considered to be only contaminated by deposition from air and not by direct contamination. The single sample from the mine area in cluster C was considered an outlier.

**Fig. 6:**
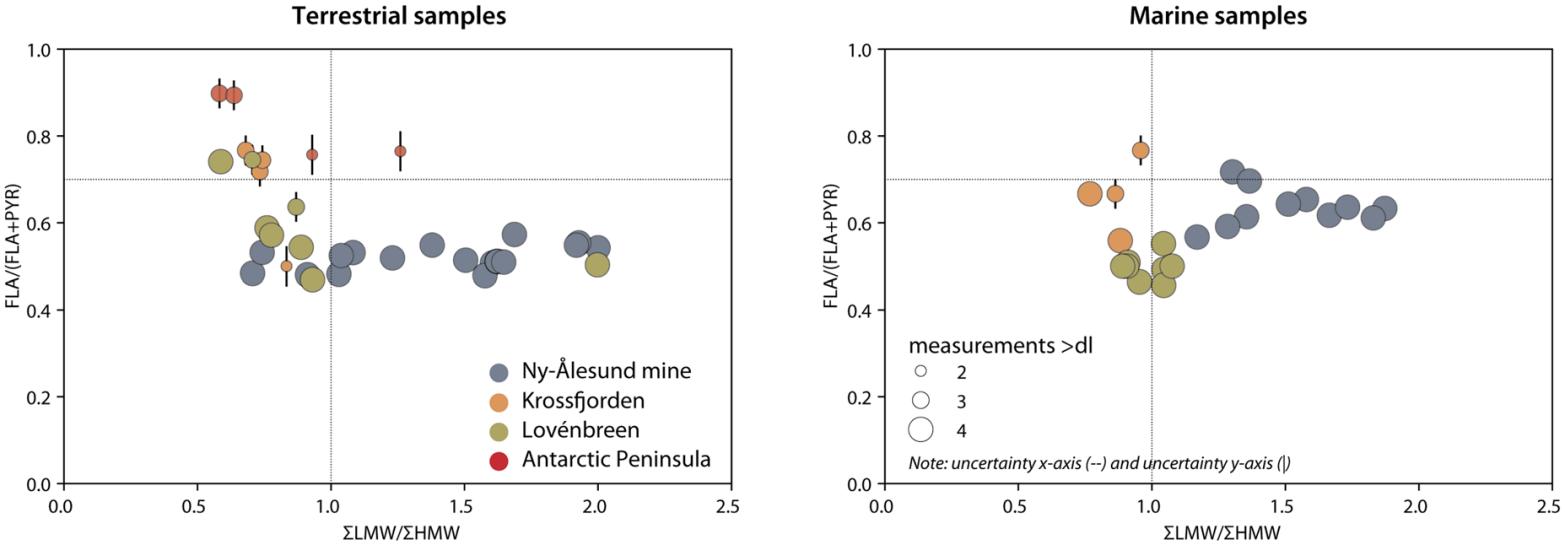
Bivariate plots of ∑LMW/∑HMW versus fluoranthene/pyrene for terrestrial and marine sediments. The size of the marker represents the number of concentrations above detection limits that are applied in the ratio calculation (see legend and ‘Materials and methods’ for more details)

In the molecular diagnostic ratio, the Krossfjorden and Antarctica terrestrial samples separated from the Ny-Ålesund mine and the Lovénbreen samples at a fluoranthene/pyrene ratio of ~ 0.7 (Fig. 6). This was a higher value than that reported by De La Roche-Torre et al. (2009) as a basis for distinguishing petrogenic origin (< 0.4) from pyrogenic fossil fuel origin (0.4–0.5), and > 0.5 for coal combustion (Tobizewski et al., 2012). The ∑LMW/∑HMW ratio categorized most mine area samples (cluster A) as petrogenic and most other samples as pyrogenic. This is consistent with our assumption that the PAHs (from anthropogenic sources) found in the reference area mostly originated from deposition by air. An important factor to consider is that our samples were collected in an area with generally very low contamination levels, in contrast to many samples sets on which the ratios reported by Tobizewski (2012) are based. It is reasonable to assume that the low PAH values found in the current study as well as environmental degradation processes were of influence to the interpretation of the observed ratios.

#### Marine sediments

The marine sediment samples separated into three clusters based on their normalized PAH profiles (Fig. 5). Cluster D, which contained all samples taken near the mine area, and cluster E, which contained the Lovénbreen samples, were both on the second level branch. All the Krossfjorden samples formed cluster F. No marine samples were collected in Antarctica. The dissimilarity between the Krossfjorden samples and those collected in the Kongsfjorden (Lovénbreen) were likely due to a different origin of the PAHs in the Krossfjorden as these samples were less likely to be affected by the mine than the Lovénbreen samples based on proximity.

Given the fact that the marine samples near the mine and those near the Lovénbreen showed a higher similarity than the terrestrial samples did from these locations, the marine sediments were thought to be more influenced by the marine environment rather than by input from the terrestrial environment alone. PAHs have a tendency to bind to organic carbon and particulates and will flow with the suspended matter in the meltwater streams into the fjord. The influence of the terrestrial run-off will therefore be highest in the shallow parts of the fjord, whereas the fjord rapidly gains depth near the coast of the mine and Lovénbreen (Husum et al., 2019). The marine samples were collected on the slope of the fjord edge, at an average depth of −26 m (mine) and −29 m (Lovénbreen). At these sites, the influence of ice-rafted detritus, material transported and deposited from icebergs or sea ice, was also observed to be relatively high compared with the Krossfjorden (Husum et al., 2019). This further supports why a weakened signal of the mine was found in the marine samples compared with the terrestrial samples.

The PAH molecular diagnostic ratios for the marine samples showed a much lower variation in both the fluoranthene/pyrene and the ∑LMW/∑HMW ratios (Fig. 6). This may suggest that the origin of the PAHs found in these samples is less variable than is the case with the terrestrial samples. The ∑LMW/∑HMW ratio clearly separated the mine samples (as petrogenic) from the other two locations (pyrogenic), although at a somewhat higher ratio (1.1 instead of 1.0) than reported in other studies (Zhang et al., 2008, Tobizewski et al., 2012). The mine samples showed higher variability in the ∑LMW/∑HMW ratio than the Krossfjorden and Lovénbreen samples which could indicate a more diverse PAH origin.

The fluoranthene/pyrene ratio showed a relatively small variation per sample location. We did not find any samples with a ratio < 0.4 which would indicate a petrogenic origin of the PAHs. The ratio 0.4–0.5 (indicating fossil fuel combustion) mainly applied to the Lovénbreen samples. Most other samples were above 0.6, which indicated coal combustion (De La Roche-Torre et al., 2009, Davis et al., 2019). The source classification based on the fluoranthene/pyrene ration was not supporting the results from the ∑LMW/∑HMW ratio. Other PAH ratios could not be applied to differentiate between the possible petrogenic and combustion sources due to the low PAH values found in these samples. A more conclusive answer based on PAH diagnostic ratios could therefore not be provided.

### Mercury–PAH relation

**Fig. 7:**
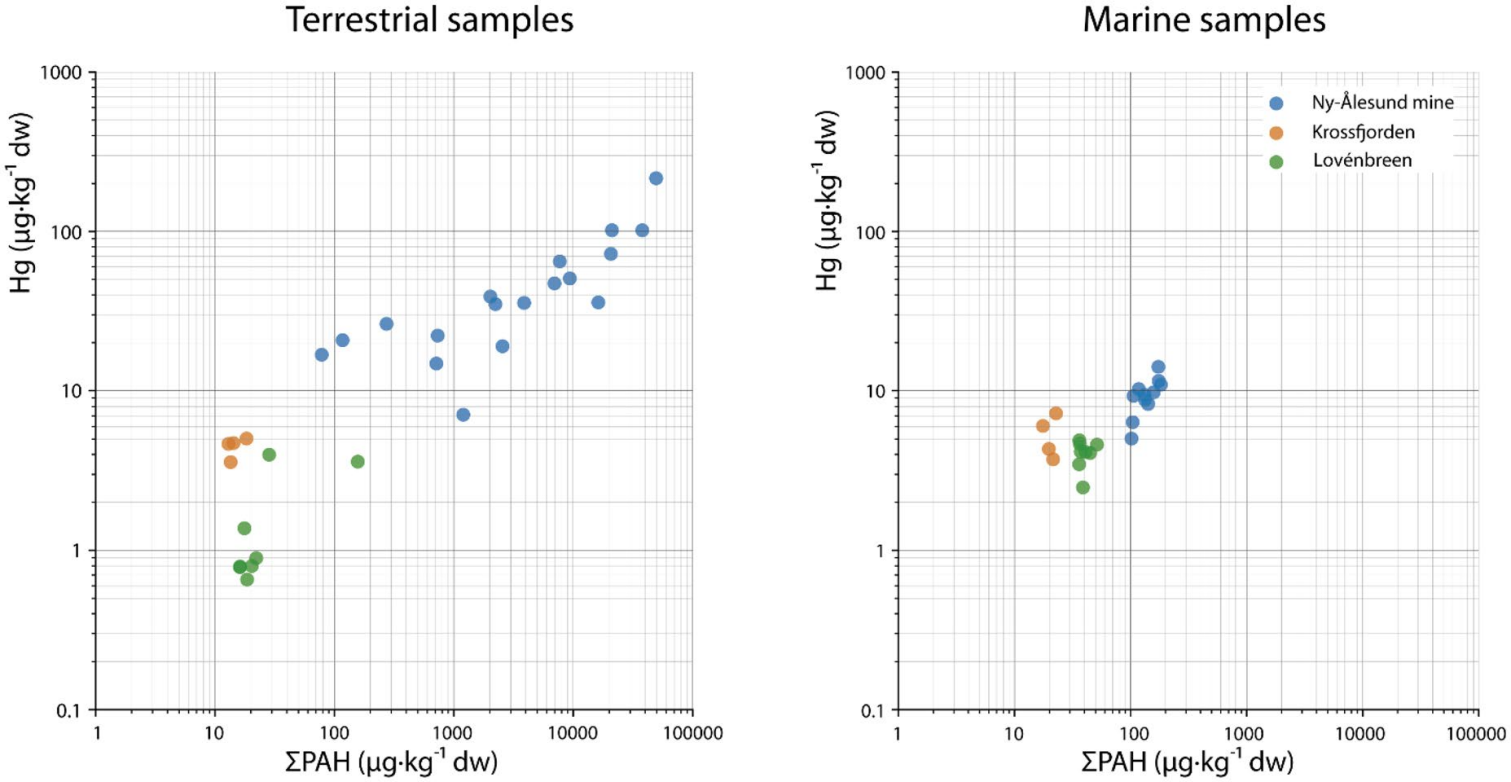
Scatter plots of Hg concentration (µg/kg dw) versus PAH (µg/kg dw) concentrations in the terrestrial samples (left**)** and in marine samples (right), collected in Kongsfjorden and Krossfjorden in 2017. The correlation factor (*R*^2^) for the terrestrial and marine samples was 0.89 and 0.91, respectively. PAH concentrations below detection limit were included as 0.5·dl

A clear relationship was observed between Hg and PAH concentrations in the terrestrial sediment samples as well as in the marine sediment samples (Fig. 7). For both the terrestrial and marine samples, we expected the Hg concentration to be elevated compared to background levels in samples for which the PAH profiles could be related to the mine. This was the case for the terrestrial samples collected at the mine site as a result of direct contamination. The correlation between PAH and Hg in the terrestrial samples collected at Lovénbreen showed a lower Hg background level in the terrestrial sediments compared with the PAH concentrations. The Hg concentrations in the terrestrial Krossfjorden samples were higher than the PAH concentrations, even though the PAH profiles and diagnostic ratios were less comparable than the mining area.

The positive correlation between the PAH and Hg levels in the Lovénbreen samples indicate that the Hg levels at this location were elevated compared with the background level. Although a clear PAH/Hg correlation was observed in the marine sediment samples, both PAHs and Hg showed much lower concentrations in these samples (Fig. 7).

### Marine biota

**Fig. 8:**
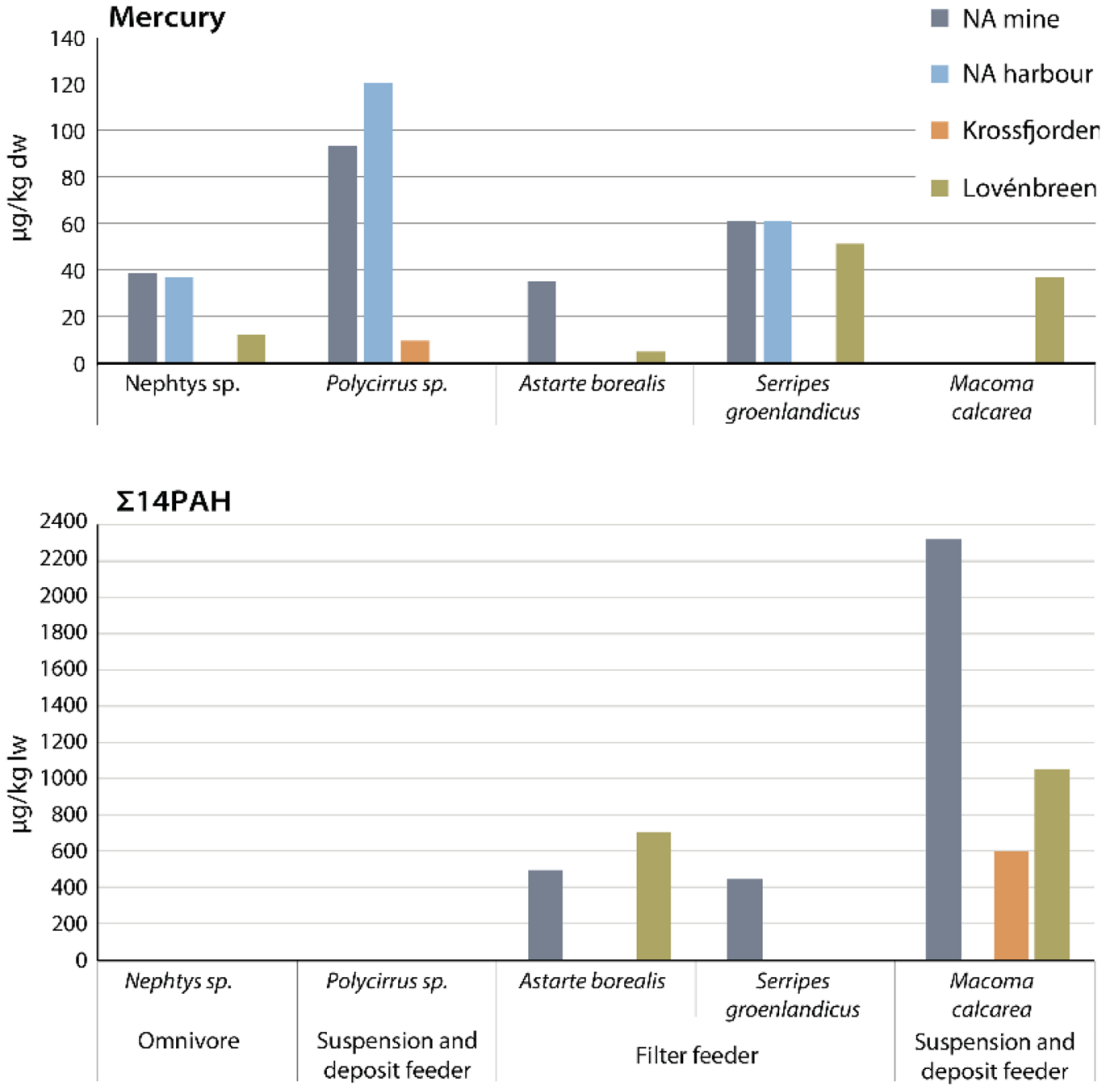
Hg (µg/kg dw) and ΣPAH (µg/kg lw) concentrations in two marine worm species (*Nephtys* sp. and *Polycirrus* sp.) and three marine shellfish species (*Astarte borealis*, *Serripes groenlandicus* and *Macoma calcarea*), collected in Kongsfjorden and Krossfjorden in July 2017

Mercury concentrations in marine biota sampled in the Kongsfjorden and Krossfjorden varied between < 10 and 120 µg/kg dw (see Table 4 in SI). Biota collected at Ny-Ålesund harbour and Ny-Ålesund mine contained 3.2–7.2 times higher Hg concentrations than biota collected at the Lovénbreen, and 9.6 times higher concentrations than those at the Krossfjorden (Fig. 8). Hg concentrations in biota in the harbour were in the same range as those near the mine (factor 0.8–1.0). One exception was *S. groenlandicus* for which Hg concentrations were in the same range for all locations in the Kongsfjorden (no data for Krossfjorden).

Marine biota from this study (< 10–120 µg/kg dw) contained similar Hg levels compared with corresponding species sampled in the Chukchi Sea (30–150 µg/kg dw; Fox et al, 2014; Fox et al, 2017), and in the Grønfjorden, Svalbard (70–170 µg/kg dw; Lebedeva et al, 2018; note: dw concentrations were based here on average dw% of the involved species as no dw was reported in the referred study). Hg concentrations in marine benthic invertebrates in the Arctic are at the lower end of what has been reported for benthic invertebrates from lower latitudes (Fox et al, 2017).

The European Commission set an Environmental Quality Standard (EQS) of 20 ng/g (µg/kg) Hg wet weight for biota to protect top predators from secondary poisoning (EC, 2008). Marine biota samples from this study contained Hg concentrations of < 1.5-–28 µg/kg Hg ww, with one sample (*Polycirrus* sp. in NA harbour) being slightly higher than the EQS. Our data are based on total Hg, whereas methylmercury is most often reported for values in biota. Methylmercury has been reported to be 30–57% of total Hg in shellfish (Pieters & Geuke, 1994).

BSAF values for Hg in the analysed biota were all above 1 (1.2–12.9) pointing at accumulation from sediment. For *M. calcarea*, only one Hg sample could be analysed in this study (Lovénbreen, BSAF of 9.1). The BSAF values for NA mine were 3.8–9.9, for Lovénbreen 1.2–12.6 and for Krossfjorden 1.8

Σ_14_PAH concentrations in marine biota of this study varied between 46 and 2340 µg/kg lw, with an average concentration of 950 ± 720 µg/kg lw Σ_14_PAH (see Table 3 in SI). Average concentrations in marine biota were higher near the mine (1100 ± 1070 µg/kg lw Σ_14_PAH) than near the Lovénbreen (890 ± 250 µg/kg lw Σ_14_PAH) and in the Krossfjorden (610 µg/kg lw Σ_14_PAH) (Fig. 8). The Σ_14_PAH concentration in the *A. borealis* sample near the Lovénbreen was 1.4 times higher than the sample at the NA mine, whereas the concentration in *M. calcarea* was 2.2 and 3.9 times higher in the sample near the mine compared to those of the Lovénbreen and Krossfjorden. PAH concentrations were highest in *M. calcarea* (4.8–5.1 times higher than those in *A. borealis* and *S. groenlandicus* near NA mine). No PAH results could be obtained for the worms due to limited sample volumes.

Marine biota from this study contained similar PAH concentrations compared with earlier published data in marine biota of the Kongsfjorden (30–9190 µg/kg lw Σ_13_PAH in *A. borealis*, *M. calcarea* and *Nephtys ciliata*; Szczybelski et al., 2016). Similar to our study, lower PAH concentrations were observed in *A. borealis* compared with *M. calcarea* (Szczybelski et al., 2016). Σ_16_PAH concentrations in *Mytilus edulis* collected in North Atlantic and sub-Arctic coastal environments (30–480 µg/kg dw; Jörundsdóttir et al., 2014) were also in the same range compared to our results (10–110 µg/kg dw).

The observed PAH concentrations in the marine biota of the Kongsfjorden and Krossfjorden were well below threshold PAH concentrations for effects in marine species, as reported for *Neanthes arenaceodentata* (Hansen et al., 2003), *Crassostrea virginica* (Hwang et al., 2008) and *Ruditapes philippinarum* (Liu et al., 2014).

**Fig. 9:**
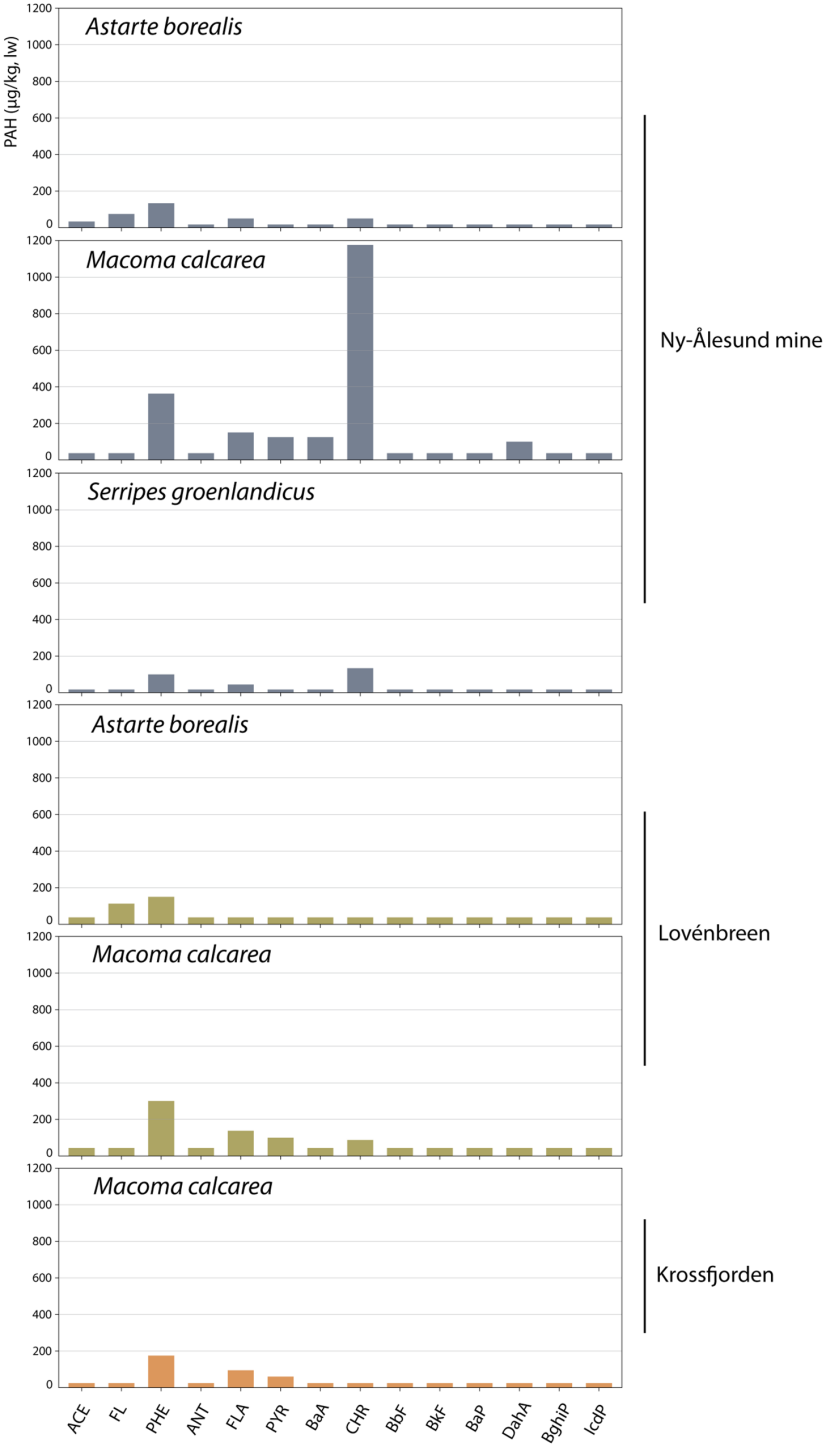
PAH profiles in bivalve samples (μg/kg, based on lw), per location, of the Kongsfjorden and Krossfjorden in July 2017. Blue = mine area, red = reference Krossfjorden, orange = reference Lovénbreen. Concentrations below the detection limit are included as 0.5 dl

PAH profiles in most biota samples showed a predominance of phenanthrene (Fig. 9). *M. calcarea* near the mine also contained a high chrysene concentration compared with the other PAH compounds. This was also found in the *S. groenlandicus* sample near the mine, though at a much lower concentration. Other PAH compounds showed variable concentrations in the samples.

The PAH profiles in the sediment and in *M. calcarea* of Lovénbreen and Krossfjorden were quite similar (Figs. 4 and 9). However, the phenanthrene fraction in sediment of NA mine was much higher than that in *M. calcarea*, whereas *M. calcarea* showed a higher fraction of chrysene compared with the sediment.

**Fig. 10:**
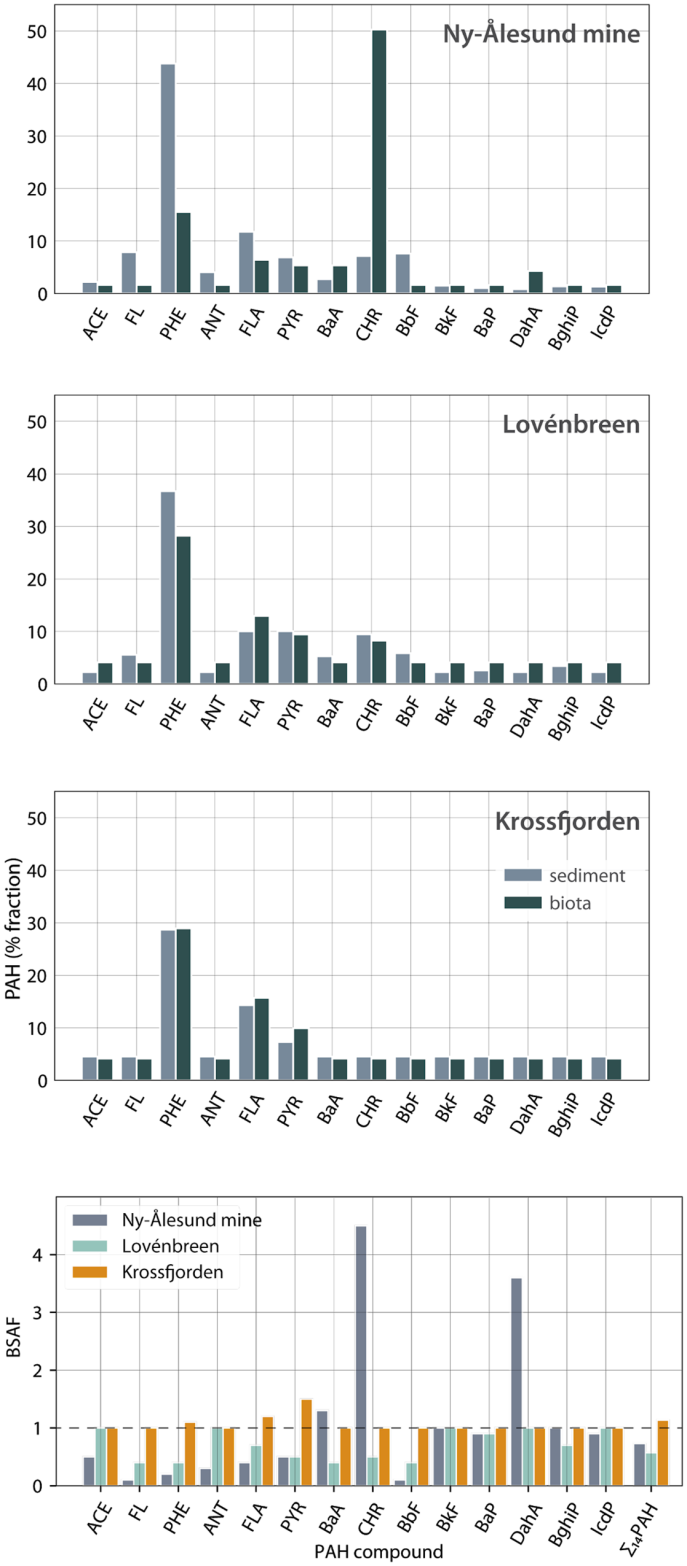
Comparison of PAH profiles (%fractions) of *Macoma calcarea* and marine sediments for samples from the Ny-Ålesund mine, Lovénbreen and Krossfjorden samples. Average values were used based on μg/kg lipid weight (biota) and μg/kg dw (sediments), including < dl as 0.5·dl. The bottom panel shows the biota sediment accumulation factor (BSAF) of *Macoma calcarea* for each PAH compound at the three locations (BSAF > 1 = concentration/accumulation, BSAF < 1 = metabolization/excretion)

A high variation in BSAF values for PAH compounds in *M. calcarea* was observed for NA mine (0.1–4.5), although most BSAF values were low (< 1) (Fig. 10, lowest panel). This was also found in earlier studies where BSAFs of PAHs were generally low (BSAF < 1) (Szczybelski et al., 2019). BSAF values for the Lovénbreen and Krossfjorden were closer together (0.4–1.0 and 1.0–1.5). Highest BSAF values were observed for dibenzo(a,h)anthracene (3.6) and chrysene (4.5) at NA mine. Bioaccumulation of chrysene was also shown in other studies in a similar species, *Macoma balthica* (current name: *Limecola balthica*) (Pikkarainen et al., 2004). The BSAF for the Σ_14_PAH was 1.1 for the Krossfjorden, 0.5 for the Lovénbreen and 0.7 for NA mine. The other two shellfish species also showed BSAF values < 1 for the Σ_14_PAH at NA mine and the Lovénbreen (0.1 and 0.4, respectively). The BSAF value for Σ_14_PAH for the Krossfjorden may be due to the fact that a lot of the concentrations were below detection limit.

The fact that the PAH profiles in marine biota and sediment near NA mine were not fully similar can be explained by the chemical characteristics of the respective PAH compounds (Lofthus et al., 2018) and biological characteristics of the benthic species (Selck et al., 2011). For tracing back the source of a terrestrial coal mine in the adjacent marine system, concentrations can therefore better be assessed in sediment samples. In case the impact of these contaminants needs to be defined, concentrations in biota provide better insight into the bioaccumulation potential of these contaminants.

## Conclusions

Based on the findings of this study, the exposure radius of Hg and PAH of the former coal mine in the Kongsfjorden was found to be relatively limited. The contaminant signal from the mine in both terrestrial and marine sediment samples was partly observed at the Lovénbreen reference site (~ 6 km from the mine), while this was absent at the Krossfjorden reference site (~ 19 km from the mine). The differences between the locations were much smaller in the marine sediments compared with the terrestrial sediments. For the biota samples the contaminant signal was even less obvious, although elevated PAH and Hg concentrations were observed in marine biota near the mine and for Hg also in the Ny-Ålesund harbour.

Clustering of PAH concentrations and PAH diagnostic ratios of the terrestrial and marine sediments showed a clear difference between the mine and the two reference sites. Also, the Lovénbreen terrestrial samples only partly clustered with the Krossfjorden samples. PAH diagnostic ratios suggested different pyrogenic sources for the Lovénbreen, compared with the Krossfjorden and Antarctic terrestrial samples.

The PAH diagnostic ratios applied in this study were based on studies conducted in areas with much higher contaminant levels. In our study, the ratios based on summed PAH (∑LMW/∑HMW) and fluoranthene/pyrene were only applicable, hereby avoiding detection limits issues. We therefore pose that the use of PAH diagnostic ratios in low contaminated areas should be only applied in combination with other methods such as hierarchical clustering. The observed low Hg and PAH background concentrations in our terrestrial sediment samples and large differences between locations on Svalbard and more generally the Arctic region, shows the need for a more in-depth study of Hg and PAH background levels in Arctic terrestrial sediments. As the organic matter may strongly influence Hg levels in these samples, it is recommended to assess Hg and PAH concentrations in samples that are both low and high in organic content.

A clear relationship was observed between PAH and Hg concentrations in the terrestrial samples collected at the mine and at the Lovénbreen site. In the Krossfjorden samples, the different relative concentrations of Hg and PAHs indicated that the mine was not the dominant source of contamination in this area. The relatively higher concentrations of mercury at this site may reflect other sources, e.g. deposition of long-range transported mercury and local geology.

Marine biota samples showed elevated Hg and PAH concentrations near the mine outflow, and for Hg also in the harbour. PAH profiles were variable and did only slightly match the characteristic PAH profile of the mine area. Accumulation from sediment was low for PAHs but considerable for Hg. Based on these observations the marine biota was considered less suitable to trace the exposure radius from a local land-based source as the historic coal mine.

In a rapidly warming Arctic changes in precipitation, snow cover, soil temperature and permafrost will inevitably lead to an increased mobility of contaminants, as well as faster bio-degradation of organic substances such as PAHs. This means that in the future increased runoff and higher availability of contaminants through leaching may enhance inputs of PAH and Hg from the terrestrial contaminated site into the marine system. Airborne deposition is not directly expected to increase, although climate-related factors can also influence atmospheric inputs from other sources.

This study shows that determining Hg and PAH concentrations in terrestrial samples and marine sediment samples is a suitable method to assess the exposure radius of a land-based coal mine and the inflow into the adjacent marine system. A follow-up study is needed to more accurately determine the edge of the exposure radius of the Ny-Ålesund coal mine by conducting a more detailed sediment sampling campaign along the Kongsfjorden, both on land and in the adjacent marine system. This would also involve improved characterisation of the natural background concentrations (of Hg as well as PAH) in the region in order to define what is and what is not an impacted area. This may also include assessing whether climate change related factors are altering the edge of the exposure radius leading to for instance increased inputs from the contaminated site further into the surrounding area.

## Supplementary Information

Below is the link to the electronic supplementary material.

Supplementary file1 (DOCX 307 KB)

## Data Availability

All data generated or analysed during this study are included in this published article and its supplementary information files.
