# Supplementary material for: Exposure radius of a local coal mine in an Arctic coastal system; correlation between PAHs and mercury as a marker for a local mercury source
Source: Environ Monit Assess. 2021 Jul 21;193(8):499. doi: 10.1007/s10661-021-09287-5 (PMC8295130; doi:10.1007/s10661-021-09287-5)
Supplement: Supplementary file 1 — Supplementary file1 (DOCX 307 KB) [file 10661_2021_9287_MOESM1_ESM.docx]

**Exposure radius of a local coal mine in an Arctic coastal system**; correlation between PAHs and mercury as a marker for a local mercury source (F. Steenhuisen & M. van den Heuvel-Greve)

**Supporting information**

**Map 1** Sample locations on the Antarctic Peninsula.

**
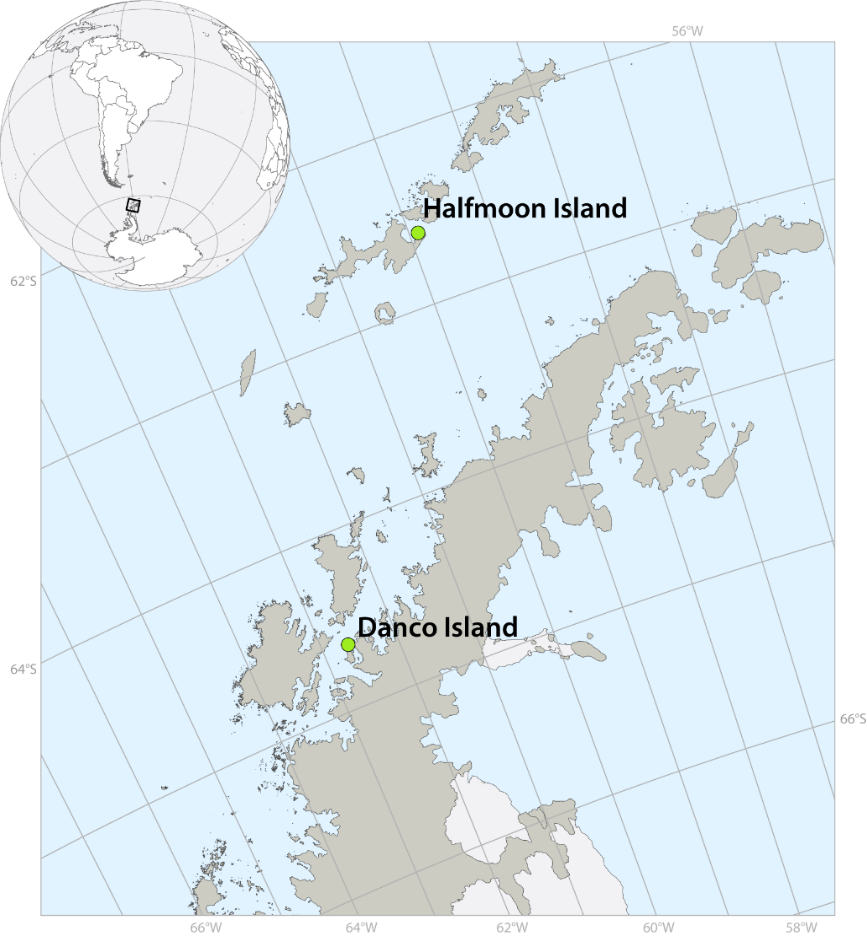
**

**Table 1** PAH compounds and used abbreviations

| Naphthalene | **NP** |
| --- | --- |
| Acenaphthene | **ACE** |
| Fluorene | **FL** |
| Phenanthrene | **PHE** |
| Anthracene | **ANT** |
| Fluoranthene | **FLA** |
| Pyrene | **PYR** |
| Benzo[a]anthracene | **BaA** |
| Chrysene | **CHR** |
| Benzo[e]pyrene | **BeP** |
| Benzo[b]fluoranthene | **BbF** |
| Benzo[k]fluoranthene | **BkF** |
| Benzo[a]pyrene | **BaP** |
| Dibenzo[a,h]anthracene | **DahA** |
| Benzo[g,h,i]perylene | **BghiP** |
| Indeno[1,2,3-c,d]pyrene | **IcdP** |

**Table 2** Sediment samples, Hg and PAH concentrations. Values below DL are indicated with <. Marine sediment samples are marked blue.

|  | Sample coordinate | |  |  |  | mg/kg ww | µg/kg ww | | | | | | | | | | | | | |
| --- | --- | --- | --- | --- | --- | --- | --- | --- | --- | --- | --- | --- | --- | --- | --- | --- | --- | --- | --- | --- |
| location | latitude | longitude | depth | dw % | TOC % | Hg | ACE | FL | PHE | ANT | FLA | PYR | BaA | CHR | BbF | BkF | BaP | DahA | BghiP | IcdP |
| Ny-Ålesund_mine | 78.9270 | 11.9684 | -13.2 | 83.5 | 3.3 | 0.0042 | 2.3 | 6.4 | 37.0 | 4.5 | 10.0 | 5.3 | 1.9 | 5.8 | 5.6 | <1.3 | <1.3 | 1.9 | 2.3 | <1.3 |
| Ny-Ålesund_mine | 78.9254 | 11.9829 | -25.7 | 68.7 | 4 | 0.0057 | 2.1 | 7.4 | 41.0 | 3.8 | 18.0 | 7.1 | 2.0 | 7.2 | 5.6 | <1.2 | <1.2 | <1.2 | <1.2 | <1.2 |
| Ny-Ålesund_mine | 78.9225 | 12.0010 | -16.8 | 72.1 | 5.3 | 0.0046 | 1.7 | 5.4 | 33.0 | 2.5 | 11.0 | 4.8 | 1.5 | 4.7 | 5.5 | 2.4 | <1.3 | <1.3 | <1.3 | <1.3 |
| Ny-Ålesund_mine | 78.9223 | 12.0165 | -28.1 | 65.4 | 0.8 | 0.0067 | 1.5 | 5.9 | 32.0 | 3.0 | 8.9 | 5.6 | 2.1 | 5.1 | 6.0 | 2.4 | <1.2 | <1.2 | 1.6 | 1.5 |
| Ny-Ålesund_mine | 78.9228 | 12.0090 | -24.9 | 64.7 | 4.6 | 0.006 | 1.8 | 5.3 | 31.0 | 2.4 | 9.9 | 5.5 | 1.6 | 3.6 | 4.4 | <1.2 | <1.2 | <1.2 | <1.2 | <1.2 |
| Ny-Ålesund_mine | 78.9227 | 12.0120 | -22.2 | 64.8 | 4.1 | 0.0057 | <1.3 | 5.6 | 46.0 | 2.7 | 10.0 | 5.8 | 1.9 | 4.6 | 5.1 | <1.3 | <1.3 | <1.3 | 1.3 | <1.3 |
| Ny-Ålesund_mine | 78.9231 | 12.0028 | -26.7 | 69.1 | 3.2 | 0.0065 | 2.1 | 7.7 | 42.0 | 3.8 | 10.0 | 6.2 | 2.6 | 6.2 | 6.6 | <1.2 | <1.2 | <1.2 | <1.3 | 1.5 |
| Ny-Ålesund_mine | 78.9236 | 11.9949 | -20.4 | 63.4 | 3.9 | 0.0073 | 3.3 | 9.7 | 52.0 | 5.7 | 11.0 | 7.0 | 3.8 | 8.6 | 6.3 | <1.3 | <1.3 | <1.3 | <1.3 | <1.3 |
| Ny-Ålesund_mine | 78.9245 | 11.9881 | -27.1 | 66.5 | 4.5 | 0.0065 | 2.9 | 9.0 | 48.0 | 5.6 | 11.0 | 6.3 | 2.9 | 7.9 | 7.9 | <1.2 | <1.2 | <1.2 | 1.3 | <1.2 |
| Ny-Ålesund_mine | 78.9271 | 11.9748 | -52 | 62.4 | 4.7 | 0.0088 | 2.6 | 9.6 | 44.0 | 3.1 | 9.7 | 6.7 | 3.8 | 10.0 | 12.0 | <1.3 | 2.7 | <1.3 | <1.3 | 2.0 |
| Ny-Ålesund_mine | 78.9239 | 11.9922 | -26 | 63.3 | 4.4 | 0.0069 | 2.2 | 8.6 | 46.0 | 4.0 | 11.0 | 8.4 | 3.8 | 11.0 | 13.0 | <1.3 | 3.5 | <1.3 | <1.3 | 2.0 |
| Ny-Ålesund_mine | 78.9116 | 11.9567 |  | 66.7 | 7.4 | 0.026 | 14.0 | 52.0 | 750.0 | 25.0 | 110.0 | 82.0 | 42.0 | 140.0 | 84.0 | 5.9 | 34.0 | <1.3 | <1.3 | 9.6 |
| Ny-Ålesund_mine | 78.9107 | 11.9532 |  | 57.1 | 4.4 | 0.015 | <1.2 | 2.7 | 55.0 | <1.2 | 15.0 | 16.0 | 5.2 | 22.0 | 13.0 | 3.2 | 7.6 | 1.4 | 6.7 | 7.1 |
| Ny-Ålesund_mine | 78.9122 | 11.9628 |  | 78.7 | 13 | 0.051 | 140.0 | 580.0 | 3200.0 | 92.0 | 320.0 | 270.0 | 240.0 | 620.0 | 310.0 | 16.0 | 120.0 | 110.0 | 34.0 | 16.0 |
| Ny-Ålesund_mine | 78.9135 | 11.9771 |  | 76.3 | 9.1 | 0.036 | 97.0 | 400.0 | 2900.0 | 75.0 | 310.0 | 250.0 | 200.0 | 600.0 | 290.0 | 15.0 | 110.0 | 26.0 | 35.0 | <12 |
| Ny-Ålesund_mine | 78.9154 | 11.9803 |  | 73.1 | 6.2 | 0.026 | 60.0 | 230.0 | 1500.0 | 58.0 | 170.0 | 140.0 | 110.0 | 320.0 | 160.0 | <14 | 49.0 | <14 | 17.0 | <14 |
| Ny-Ålesund_mine | 78.9184 | 11.9641 |  | 69.2 | 51.1 | 0.05 | 320.0 | 770.0 | 6700.0 | 680.0 | 690.0 | 670.0 | 720.0 | 1400.0 | 880.0 | 59.0 | 580.0 | 280.0 | 520.0 | 41.0 |
| Ny-Ålesund_mine | 78.9184 | 11.9641 |  | 69.1 | 20.1 | 0.035 | 170.0 | 450.0 | 2900.0 | 300.0 | 340.0 | 370.0 | 340.0 | 550.0 | 380.0 | 33.0 | 270.0 | 140.0 | 200.0 | 31.0 |
| Ny-Ålesund_mine | 78.9175 | 11.9590 |  | 67 | 21.8 | 0.024 | 290.0 | 660.0 | 4800.0 | 550.0 | 560.0 | 530.0 | 590.0 | 1000.0 | 640.0 | 48.0 | 470.0 | 350.0 | 350.0 | 29.0 |
| Ny-Ålesund_mine | 78.9164 | 11.9515 |  | 77.7 | 45.8 | 0.079 | 430.0 | 1100.0 | 7800.0 | 800.0 | 930.0 | 890.0 | 1500.0 | 1300.0 | 980.0 | <15 | 630.0 | <15 | <15 | <15 |
| Ny-Ålesund_mine | 78.9156 | 11.9489 |  | 43.3 | 22.2 | 0.044 | 430.0 | 1100.0 | 7800.0 | 800.0 | 930.0 | 890.0 | 1500.0 | 1300.0 | 980.0 | <15 | 630.0 | <15 | <15 | <15 |
| Ny-Ålesund_mine | 78.9118 | 11.9357 |  | 77.3 | 2.6 | 0.013 | <1.2 | <1.2 | 23.0 | <1.2 | 4.9 | 5.3 | 2.6 | 10.0 | 2.6 | <1.2 | <1.2 | <1.2 | 6.8 | 1.7 |
| Ny-Ålesund_mine | 78.9125 | 11.9319 |  | 72.3 | 3.1 | 0.015 | <1.3 | 4.3 | 31.0 | <1.3 | 6.7 | 7.2 | 2.6 | 15.0 | 2.1 | <1.3 | <1.3 | <1.3 | 8.8 | 3.7 |
| Ny-Ålesund_mine | 78.9138 | 11.9384 |  | 64.9 | 25.7 | 0.14 | 790.0 | 2000.0 | 8900.0 | 1200.0 | 4200.0 | 3700.0 | 2400.0 | 3000.0 | 1300.0 | <70 | 2700.0 | <70 | <71 | 1800.0 |
| Ny-Ålesund_mine | 78.9156 | 11.9356 |  | 78.9 | 4 | 0.015 | 14.0 | 37.0 | 1100.0 | <13 | 170.0 | 140.0 | 130.0 | 330.0 | 50.0 | <13 | <13 | <13 | <13 | 14.0 |
| Ny-Ålesund_mine | 78.9175 | 11.9443 |  | 74.4 | 4.5 | 0.026 | 33.0 | 94.0 | 650.0 | 29.0 | 170.0 | 150.0 | 150.0 | 200.0 | 14.0 | 14.0 | 40.0 | <13 | 70.0 | 40.0 |
| Ny-Ålesund_mine | 78.9175 | 11.9443 |  | 76.7 | 3.5 | 0.017 | <14 | <14 | 250.0 | <14 | 43.0 | 39.0 | 69.0 | 75.0 | 14.0 | <14 | <14 | <14 | 23.0 | <14 |
| Ny-Ålesund_mine | 78.9187 | 11.9516 |  | 87.8 | 3.2 | 0.013 | <13 | 33.0 | 280.0 | <13 | 55.0 | 51.0 | 48.0 | 85.0 | <13 | <13 | <13 | <13 | 20.0 | 16.0 |
| Ny-Ålesund_mine | 78.9199 | 11.9581 |  | 80.2 | 3.2 | 0.0057 | <15 | 50.0 | 510.0 | 22.0 | 54.0 | 52.0 | 80.0 | 99.0 | 41.0 | <15 | 17.0 | <15 | <15 | <15 |
| Krossfjorden | 79.1476 | 11.5620 |  | 77.2 | 1.6 | 0.0036 | <1.2 | <1.2 | 2.2 | <1.2 | <1.2 | <1.2 | <1.2 | <1.2 | <1.2 | <1.2 | <1.2 | <1.2 | <1.2 | <1.2 |
| Krossfjorden | 79.1405 | 11.5210 |  | 73.5 | 1.7 | 0.0037 | <1.4 | <1.4 | 2.8 | <1.4 | 2.3 | <1.4 | <1.4 | <1.4 | <1.4 | <1.4 | <1.4 | <1.4 | <1.4 | <1.4 |
| Krossfjorden | 79.1403 | 11.5383 |  | 75.4 | 1.2 | 0.0027 | <1.1 | <1.1 | 2.2 | <1.1 | 1.4 | <1.1 | <1.1 | <1.1 | <1.1 | <1.1 | <1.1 | <1.1 | <1.1 | <1.1 |
| Krossfjorden | 79.1428 | 11.5603 |  | 74.2 | 1.4 | 0.0035 | <1.1 | <1.1 | 2.4 | <1.1 | 1.6 | <1.1 | <1.1 | <1.1 | <1.1 | <1.1 | <1.1 | <1.1 | <1.1 | <1.1 |
| Krossfjorden | 79.1544 | 11.6342 | -8 - -12m | 69.5 | 4.1 | 0.0042 | <1.3 | <1.3 | 3.1 | <1.3 | 1.3 | <1.3 | <1.3 | <1.3 | <1.3 | <1.3 | <1.3 | <1.3 | <1.3 | <1.3 |
| Krossfjorden | 79.1552 | 11.6410 | -8 - -12m | 78.5 | 3.3 | 0.0034 | <1.4 | <1.4 | 4.8 | <1.4 | 2.3 | <1.4 | <1.4 | <1.4 | <1.4 | <1.4 | <1.4 | <1.4 | <1.4 | <1.4 |
| Krossfjorden | 79.1556 | 11.6355 | -8 - -12m | 75.1 | 3.7 | 0.0028 | <1.3 | <1.3 | 4.5 | <1.3 | 3.0 | 1.5 | <1.3 | <1.3 | <1.3 | <1.3 | <1.3 | <1.3 | <1.3 | <1.3 |
| Krossfjorden | 79.1526 | 11.6333 | -8 - -12m | 69 | 3.5 | 0.005 | <1.4 | <1.4 | 4.6 | <1.4 | 1.9 | 1.5 | <1.4 | <1.4 | <1.4 | <1.4 | <1.4 | <1.4 | <1.4 | <1.4 |
| Lovénbreen | 78.9042 | 12.1562 |  | 78.3 | 0.5 | 0.0007 | <1.3 | <1.3 | 5.6 | <1.3 | 2.5 | 2.1 | <1.3 | <1.3 | <1.3 | <1.3 | <1.3 | <1.3 | <1.3 | <1.3 |
| Lovénbreen | 78.9014 | 12.1514 |  | 76.2 | 0.5 | 0.0005 | <1.3 | <1.3 | 3.6 | <1.3 | 2.0 | 1.4 | <1.3 | <1.3 | <1.3 | <1.3 | <1.3 | <1.3 | <1.3 | <1.3 |
| Lovénbreen | 78.8999 | 12.1448 |  | 75.3 | 0.5 | 0.003 | <1.2 | <1.2 | 7.6 | <1.2 | 2.3 | 2.6 | <1.2 | 1.5 | 1.3 | <1.2 | <1.2 | <1.2 | 1.3 | <1.2 |
| Lovénbreen | 78.8984 | 12.1442 |  | 83.2 | 0.7 | 0.003 | 4.1 | 13.0 | 58.0 | 7.8 | 8.0 | 7.9 | 3.7 | 9.3 | 8.2 | <1.2 | 3.2 | <1.2 | 3.8 | 2.4 |
| Lovénbreen | 78.9025 | 12.0880 |  | 75.8 | 0.4 | 0.0006 | <1.3 | <1.3 | 2.6 | <1.3 | 1.9 | <1.3 | <1.3 | <1.3 | <1.3 | <1.3 | <1.3 | <1.3 | <1.3 | <1.3 |
| Lovénbreen | 78.9042 | 12.0897 |  | 75.2 | 0.3 | 0.0006 | <1.3 | <1.3 | 3.8 | <1.3 | 2.0 | 1.5 | <1.3 | <1.3 | <1.3 | <1.3 | <1.3 | <1.3 | 1.5 | <1.3 |
| Lovénbreen | 78.9059 | 12.0955 |  | 79.9 | 0.4 | 0.0011 | <1.2 | <1.2 | 4.2 | <1.2 | 2.1 | 1.2 | <1.2 | <1.2 | <1.2 | <1.2 | <1.2 | <1.2 | <1.2 | <1.2 |
| Lovénbreen | 78.9077 | 12.1047 |  | 76.4 | 0.5 | 0.0006 | <1.4 | <1.4 | <3.9 | <1.4 | 2.0 | <1.4 | <1.4 | <1.4 | <1.4 | <1.4 | <1.4 | <1.4 | <1.4 | <1.4 |
| Lovénbreen | 78.9049 | 12.1572 | -11.8 | 75.9 | 2 | 0.0031 | <1.2 | 2.1 | 13.0 | <1.2 | 3.3 | 3.4 | 1.9 | 3.1 | 2.1 | <1.2 | <1.2 | <1.2 | 1.5 | <1.2 |
| Lovénbreen | 78.9053 | 12.1557 | -23.9 | 76.5 | 2.2 | 0.0019 | <1.3 | 2.1 | 10.0 | <1.3 | 2.6 | 3.0 | 1.8 | 2.9 | 1.8 | <1.3 | <1.3 | <1.3 | 1.6 | <1.3 |
| Lovénbreen | 78.9051 | 12.1541 | -26 | 68.4 | 2.1 | 0.0032 | <1.4 | <1.4 | 9.1 | <1.4 | 2.9 | 2.8 | <1.4 | 2.3 | 1.5 | <1.4 | <1.4 | <1.4 | <1.4 | <1.4 |
| Lovénbreen | 78.9056 | 12.1522 | -40 | 67.2 | 2.3 | 0.0033 | <1.2 | 1.5 | 9.1 | <1.2 | 2.1 | 2.5 | 1.4 | 2.3 | 1.2 | <1.2 | <1.2 | <1.2 | <1.2 | <1.2 |
| Lovénbreen | 78.9090 | 12.1197 | -30.4 | 69.5 | 1.8 | 0.0029 | <1.3 | <1.3 | 9.7 | <1.3 | 2.6 | 2.6 | 1.6 | 2.5 | 1.6 | <1.3 | <1.3 | <1.3 | <1.3 | <1.3 |
| Lovénbreen | 78.9088 | 12.1217 | -30.3 | 69.1 | 1.8 | 0.0024 | <1.4 | 1.6 | 9.0 | <1.4 | 3.2 | 2.6 | <1.4 | 2.2 | <1.4 | <1.4 | <1.4 | <1.4 | <1.4 | <1.4 |
| Lovénbreen | 78.9090 | 12.1216 | -34.4 | 71.5 | 2.1 | 0.0033 | <1.2 | 2.1 | 13.0 | <1.2 | 3.4 | 3.4 | 2.3 | 3.6 | 3.0 | <1.2 | 1.4 | <1.2 | 1.5 | <1.2 |
| Lovénbreen | 78.9091 | 12.1205 | -36.2 | 70 | 1.9 | 0.0029 | <1.2 | 2.0 | 11.0 | <1.2 | 2.7 | 2.7 | 1.7 | 2.7 | 1.6 | <1.2 | <1.2 | <1.2 | <1.2 | <1.2 |
| Ny-Ålesund_mine  Coal sample | 78.9154 | 11.9803 |  | 98 | 84.6 | 0.13 | 360.0 | 950.0 | 4600.0 | 260.0 | 300.0 | 370.0 | 1000.0 | 560.0 | 420.0 | <26 | 260.0 | 240.0 | 29.0 | <26 |
| Antarctic Peninsula | -64.4354 | -62.3603 |  | 84.8 | 3.8 | 0.062 | <0.8 | <0.8 | <5.5 | <0.8 | <2.8 | <0.9 | <0.8 | <0.8 | <0.8 | <0.8 | <0.8 | <0.8 | <0.8 | <0.8 |
| Antarctic Peninsula | -64.4351 | -62.3603 |  | 91.2 | 1.9 | 0.011 | <0.7 | 1.6 | <5.1 | <0.7 | <2.6 | <0.8 | <0.7 | <0.7 | <0.7 | <0.8 | <0.7 | <0.7 | <0.8 | <0.8 |
| Antarctic Peninsula | -62.3544 | -59.5411 |  | 73.2 | 3.5 | 0.015 | <0.5 | <0.5 | <3.4 | <0.5 | 2.1 | <0.5 | <0.5 | <0.5 | <0.5 | <0.5 | <0.5 | <0.5 | <0.5 | <0.5 |

**Table 3.** PAH concentrations in biota samples (µg/kg ww) collected in the Kongsfjorden and Krossfjorden in July 2017. Dw = dry weight. Concentrations in shell fish species were based on tissue without the shell.

| **Species** | **Tissue- type** | **Location** | **n** | **Length (mm)** | **Weight (g)** | **dw %** | **Lipid (BD )%** | **ACE** | **FL** | **PHE** | **ANT** | **FLA** | **PYR** | **BaA** | **CHR** | **BbF** | **BkF** | **BaP** | **DahA** | **BghiP** | **IcdP** |
| --- | --- | --- | --- | --- | --- | --- | --- | --- | --- | --- | --- | --- | --- | --- | --- | --- | --- | --- | --- | --- | --- |
| *Astarte borealis* | Whole organism | Ny-Ålesund mine | 49 | 24.1 ±8.7 | 4.5 ±5.1 | 15.9 | 1.2 | 0.4 | 0.9 | 1.6 | <0.4 | 0.6 | <0.4 | <0.4 | 0.6 | <0.4 | <0.4 | <0.4 | <0.4 | <0.4 | <0.4 |
| *Serripes groenlandicus* | Whole organism | Ny-Ålesund mine | 4 | 40.4 ±16.2 | 23.2 ±20.3 | 15.1 | 0.9 | <0.3 | <0.3 | 0.9 | <0.3 | 0.4 | <0.3 | <0.3 | 1.2 | <0.3 | <0.3 | <0.3 | <0.3 | <0.3 | <0.3 |
| *Macoma calcarea* | Whole organism | Ny-Ålesund mine | 17 | 24.1 ±6.3 | 2.7 ± 2.0 | 13.8 | 0.8 | <0.6 | <0.6 | 2.9 | <0.6 | 1.2 | 1.0 | 1.0 | 9.4 | <0.6 | <0.6 | <0.6 | 0.8 | <0.6 | <0.6 |
| *Macoma calcarea* | Whole organism | Krossfjord | 30 | 18.1 ± 5.2 | 1.0 ± 0.8 | 19.6 | 2 | <1.0 | <1.0 | 3.5 | <1.0 | 1.9 | 1.2 | <1.0 | <1.0 | <1.0 | <1.0 | <1.0 | <1.0 | <1.0 | <1.0 |
| *Macoma calcarea* | Whole organism | Lovénbreen | 32 | 21.0 ± 5.9 | 1.4 ± 1.3 | 12.5 | 0.8 | <0.7 | <0.7 | 2.4 | <0.7 | 1.1 | 0.8 | <0.7 | 0.7 | <0.7 | <0.7 | <0.7 | <0.7 | <0.7 | <0.7 |
| *Astarte borealis* | Whole organism | Lovénbreen | 19 | 25.8 ± 10.7 | 6.0 ± 6.5 | 15 | 0.8 | <0.6 | 0.9 | 1.2 | <0.6 | <0.6 | <0.6 | <0.6 | <0.6 | <0.6 | <0.6 | <0.6 | <0.6 | <0.6 | <0.6 |

**Table 4.** Total mercury concentrations in biota samples (mg/kg ww) collected in the Kongsfjorden and Krossfjorden in July 2017. Dw = dry weight. Concentrations in shell fish species were based on tissue without the shell.

| **Species** | **Tissue type** | **Location** | **n** | **Length (mm)** | **Weight (g)** | **Monster type** | **Monstercode** | **Date** | **dw %** | **Hg (ww)** |
| --- | --- | --- | --- | --- | --- | --- | --- | --- | --- | --- |
| *Nephtys* sp. | Whole organism | Ny-Ålesund mine | 1 | - | 3.6 | Vlees | I | 19/07/2017 | 19.9 | 0.0077 |
| *Nephtys* sp. | Whole organism | Lovénbreen | 10 | - | 0.5 ± 0.3 | Vlees | X | 26/07/2017 | 19.9 | 0.0024 |
| Nephtys sp. | Whole organism | Ny-Ålesund harbour | 1 | - | 2.6 | Vlees | XVI | 26/07/2017 | 27.2 | 0.0100 |
| Tube worm | Whole organism | Ny-Ålesund mine | 3 | - | 0.9 | Vlees | II | 19/07/2017 | 18.2 | 0.0170 |
| Tube worm | Whole organism | Krossfjord | 2 | - | 2.5 ± 2.8 | Vlees | IX | 24/07/2017 | 20.6 | 0.0020 |
| Tube worm | Whole organism | Ny-Ålesund harbour | 4 | - | 2.9 ± 1.4 | Vlees | XV | 26/07/2017 | 23.2 | 0.0280 |
| *Astarte borealis* | Whole organism | Ny-Ålesund mine | 48 | 24.9 ± 9.5 | 4.9 ± 5.7 | Vlees | III | 19/07/2017 | 16.4 | 0.0058 |
| *Astarte borealis* | Whole organism | Lovénbreen | 18 | 25.3 ± 11.2 | 6.2 ± 7.7 | Vlees | XI | 26/07/2017 | 15.3 | <0.0015 |
| *Serripes groenlandicus* | Whole organism | Ny-Ålesund mine | 3 | 49.9 ± 8.2 | 33.3 ± 18.6 | Vlees | V | 19/07/2017 | 16.3 | 0.0100 |
| *Serripes groenlandicus* | Whole organism | Lovénbreen | 2 | 45.5 ± 14.8 | 25.9 ± 23.0 | Vlees | XIII | 26/07/2017 | 14.8 | 0.0076 |
| *Serripes groenlandicus* | Whole organism | Ny-Ålesund harbour | 1 | 65.1 | 72.5 | Vlees | XIV | 26/07/2017 | 16.3 | 0.0100 |
| *Macoma calcarea* | Whole organism | Lovénbreen | 30 | 21.0 ± 6.1 | 1.4 ± 1.2 | Vlees | XII | 26/07/2017 | 14.1 | 0.0052 |
